# Prediction of Prophages and Their Host Ranges in Pathogenic and Commensal *Neisseria* Species

**DOI:** 10.1128/msystems.00083-22

**Published:** 2022-04-14

**Authors:** Giulia Orazi, Alan J. Collins, Rachel J. Whitaker

**Affiliations:** a Carl R. Woese Institute for Genomic Biology, University of Illinois at Urbana-Champaign, Urbana, Illinois, USA; b Department of Microbiology, University of Illinois at Urbana-Champaign, Urbana, Illinois, USA; Pacific Northwest National Laboratory

**Keywords:** comparative genomics, bacteriophages, *Neisseria*, CRISPR

## Abstract

The genus *Neisseria* includes two pathogenic species, N. gonorrhoeae and N. meningitidis, and numerous commensal species. *Neisseria* species frequently exchange DNA with one another, primarily via transformation and homologous recombination and via multiple types of mobile genetic elements (MGEs). Few *Neisseria* bacteriophages (phages) have been identified, and their impact on bacterial physiology is poorly understood. Furthermore, little is known about the range of species that *Neisseria* phages can infect. In this study, we used three virus prediction tools to scan 248 genomes of 21 different *Neisseria* species and identified 1,302 unique predicted prophages. Using comparative genomics, we found that many predictions are dissimilar from prophages and other MGEs previously described to infect *Neisseria* species. We also identified similar predicted prophages in genomes of different *Neisseria* species. Additionally, we examined CRISPR-Cas targeting of each *Neisseria* genome and predicted prophage. While CRISPR targeting of chromosomal DNA appears to be common among several *Neisseria* species, we found that 20% of the prophages we predicted are targeted significantly more than the rest of the bacterial genome in which they were identified (i.e., backbone). Furthermore, many predicted prophages are targeted by CRISPR spacers encoded by other species. We then used these results to infer additional host species of known *Neisseria* prophages and predictions that are highly targeted relative to the backbone. Together, our results suggest that we have identified novel *Neisseria* prophages, several of which may infect multiple *Neisseria* species. These findings have important implications for understanding horizontal gene transfer between members of this genus.

**IMPORTANCE** Drug-resistant Neisseria gonorrhoeae is a major threat to human health. Commensal *Neisseria* species are thought to serve as reservoirs of antibiotic resistance and virulence genes for the pathogenic species N. gonorrhoeae and N. meningitidis. Therefore, it is important to understand both the diversity of mobile genetic elements (MGEs) that can mediate horizontal gene transfer within this genus and the breadth of species these MGEs can infect. In particular, few bacteriophages (phages) are known to infect *Neisseria* species. In this study, we identified a large number of candidate phages integrated in the genomes of commensal and pathogenic *Neisseria* species, many of which appear to be novel phages. Importantly, we discovered extensive interspecies targeting of predicted phages by *Neisseria* CRISPR-Cas systems, which may reflect their movement between different species. Uncovering the diversity and host range of phages is essential for understanding how they influence the evolution of their microbial hosts.

## INTRODUCTION

The genus *Neisseria* includes the human pathogens N. gonorrhoeae and N. meningitidis, as well as a multitude of diverse commensal species that colonize mucosal surfaces of humans and animals (1). Because of the extensive spread of antibiotic resistance among strains of N. gonorrhoeae, infections caused by this pathogen are becoming increasingly difficult to treat (2). Consequently, the WHO and CDC consider N. gonorrhoeae a high-priority and urgent threat (3, 4). While resistance to frontline treatment is rare in N. meningitidis, penicillin-resistant strains have been recently detected in multiple countries and may pose an emerging threat (5–7).

*Neisseria* species are naturally competent and frequently exchange DNA with one other via transformation and homologous recombination (8–11). Mobile genetic elements (MGEs), such as plasmids, genetic islands, and bacteriophages (phages), can also mobilize genetic material and are powerful forces in shaping bacterial evolution (12–17). Phages are incredibly abundant and can profoundly influence the fitness and virulence of their bacterial hosts, particularly when integrated into the bacterial chromosome as prophages (18–23). For example, the filamentous prophage MDAΦ promotes attachment of N. meningitidis to epithelial cell monolayers (21) and is associated with the ability of this pathogen to cause invasive disease (20).

In contrast to many highly studied *Gammaproteobacteria* prophages, few have been identified and characterized in the *Betaproteobacteria* (24–26). *Neisseria* prophages have been identified primarily in N. gonorrhoeae and N. meningitidis and consist of a small number of filamentous (27–30) and double-stranded DNA (dsDNA) prophages (31), including Mu-like prophages (24, 32–35). With the exception of MDAΦ, the impact of phages on *Neisseria* biology and pathogenicity remains poorly understood (16). Furthermore, few studies have investigated the host ranges of *Neisseria* phages (36, 37).

Microbes can defend themselves against phages and other MGEs using a variety of systems. One such system is CRISPR-Cas, which is composed of clustered regularly interspaced short palindromic repeat (CRISPR) arrays and CRISPR-associated (Cas) proteins. Importantly, sequence identity between the spacer and the MGE is required for immunity, which means that CRISPR arrays contain a record of previous encounters with MGEs in the sequences of their spacers. Therefore, this historical record can be used to infer the bacterial hosts of viruses (38–42). Approximately 40% of N. meningitidis genomes encode type II-C CRISPR arrays (43, 44), and multiple putative CRISPR systems have been identified in several commensal species (44–46). In contrast, no functional CRISPR systems have been identified in N. gonorrhoeae (44, 45).

In this study, we sought to uncover novel *Neisseria* phage diversity. We used bioinformatic virus prediction tools to scan publicly available genomes of pathogenic and commensal *Neisseria* species for prophages. Using comparative genomics, we found that many of these predictions are dissimilar from previously identified *Neisseria* MGEs and are potential targets of CRISPR-Cas systems. Finally, we used interspecies CRISPR targeting of known and predicted prophages to infer whether they may infect multiple different *Neisseria* species.

## RESULTS

### Predicting prophages in genomes of pathogenic and commensal *Neisseria* species.

**FIG 1:**
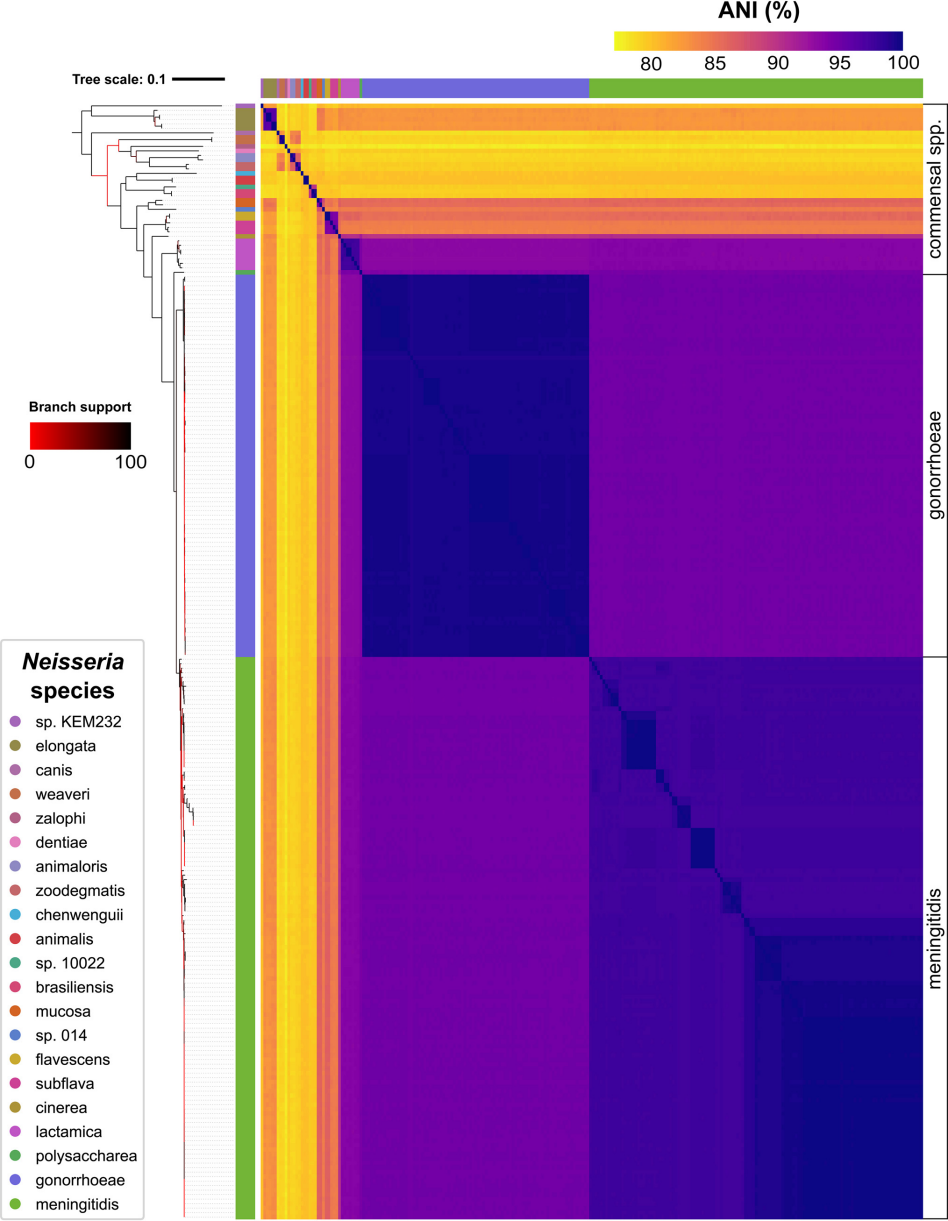
Relationships between bacterial genomes used in this study. Maximum likelihood ribosomal multilocus sequence typing (rMLST) tree of the smaller set of high-quality *Neisseria* genomes (described in Table S1, tab A) and a heatmap of pairwise average nucleotide identity (ANI) values between each genome. Ribosomal gene sequences were identified and concatenated using PubMLST and used to create a phylogenetic tree using RAxML. The tree was visualized with iTOL and rooted using midpoint rooting. The species of each genome is indicated by the vertical color strip to the right of the tree (and the identical horizontal color strip above the heatmap), where each color represents a different species as defined in the *Neisseria* species key. The order of species in the tree is the same as the order shown in the key. Bootstrap support is indicated by the color of each branch, where red indicates low support as defined in the branch support key. The tree file is provided in Data set S1, tab 1. ANI values were calculated using FastANI and are represented as a color gradient as indicated in the ANI (%) key.

To search for prophages, we compiled a data set of 248 publicly available high-quality genome assemblies of N. gonorrhoeae, N. meningitidis, and 19 commensal species that were obtained from GenBank (47, 48) (see Materials and Methods, Table S1, tab A. The relationships between the genomes in this data set are shown in a phylogenetic tree based on ribosomal gene sequences and in a heatmap of the average nucleotide identity (ANI) between each pair of genomes (Fig. 1). The phylogeny presented here is consistent with previously reported relationships between *Neisseria* species (49).

10.1128/msystems.00083-22.7 TABLE S1 Sequences used in this study. (A) Smaller set of *Neisseria* genome assemblies used in this study to predict prophages. (B) Prophages previously identified in *Neisseria* genomes. (C) Illumina reads associated with *Neisseria* genome assemblies from the smaller data set. (D) *Neisseria* plasmids and the gonococcal genetic island. (E) Larger set of *Neisseria* genome assemblies used in this study to identify CRISPR arrays. (F) Number of genomes of each species included in the small and large data sets. Download Table S1, XLSX file, 0.2 MB. Copyright © 2022 Orazi et al. 2022 Orazi et al. https://creativecommons.org/licenses/by/4.0/ This content is distributed under the terms of the Creative Commons Attribution 4.0 International license.

10.1128/msystems.00083-22.9 DATA SET S1 Data included in each tab. (1) Bacterial genome phylogenetic tree file. (2) Predicted prophages that cluster with known *Neisseria* plasmids and gonococcal genetic islands by hierarchical clustering. (3) vConTACT subcluster information and memberships. (4 to 6) vConTACT network files. (7) CRISPR arrays identified in each *Neisseria* genome. (8) *Neisseria* CRISPR repeat sequences and associated subtypes. (9) *Neisseria* CRISPR spacer sequences. (10) CRISPR targeting densities of known and predicted prophages compared to backbone genome targeting density. (11) Dereplicated prophages with CRISPR targeting densities that are significantly higher than those of the bacterial genome. (12) vConTACT subclusters that include significantly targeted predicted prophages. (13) Identical spacers identified in genomes of different *Neisseria* species. (14) Matches between spacers encoded by other bacterial taxa and predicted *Neisseria* prophages. Download Data Set S1, XLSX file, 3.3 MB. Copyright © 2022 Orazi et al. 2022 Orazi et al. https://creativecommons.org/licenses/by/4.0/ This content is distributed under the terms of the Creative Commons Attribution 4.0 International license.

We used three bioinformatic tools to predict prophages in the above-described set of genomes, PhiSpy (50), VirSorter2 (51), and Seeker (52) (see Materials and Methods for the rationale used to select these tools). In total, we obtained 2,050 predicted prophages (Table S2, tab A).

10.1128/msystems.00083-22.8 TABLE S2 Information about predicted prophages. (A) Prophages predicted in this study. (B) Dereplication of predicted prophages at 95% length aligned. (C) Comparison of clustering and CRISPR targeting of predicted prophages between tools. (D) Inferred additional *Neisseria* host species of known prophages and significantly targeted predictions. Download Table S2, XLSX file, 0.1 MB. Copyright © 2022 Orazi et al. 2022 Orazi et al. https://creativecommons.org/licenses/by/4.0/ This content is distributed under the terms of the Creative Commons Attribution 4.0 International license.

We assessed whether the tools described above could identify nine previously described prophages in N. gonorrhoeae FA 1090 (27, 29, 31) and four in N. meningitidis Z2491 (27, 28, 32) (Table S1, tab B). Combined, the three tools predicted 6/9 known, intact prophages in FA 1090 (Fig. S1A) and 2/4 in Z2491 (Fig. S1B). Our results are consistent with previous observations that several tools have difficulty predicting *Neisseria* prophages (53).

10.1128/msystems.00083-22.1 FIG S1 Comparison of known *Neisseria* prophages to predictions made by three bioinformatic tools. (A and B) Visualizations of locations of known and predicted prophages made using SnapGene software (from Insightful Science; available at snapgene.com) and modified. PhiSpy, VirSorter2, or Seeker were used to predict prophages in two bacterial genomes, N. gonorrhoeae FA10 (A) and N. meningitidis Z2491 (B). Known dsDNA prophages are shown in dark gray, known filamentous prophages in light gray, and predictions are shown in different colors as indicated in the key. Known *Neisseria* prophages are described in Table S1, tab B, and information about each prediction is provided in Table S2, tab A. Download FIG S1, TIF file, 1.3 MB. Copyright © 2022 Orazi et al. 2022 Orazi et al. https://creativecommons.org/licenses/by/4.0/ This content is distributed under the terms of the Creative Commons Attribution 4.0 International license.

None of the above-described tools correctly identified known *Neisseria* filamentous prophages (light gray, Fig. S1); they were either missed entirely or combined with an adjacent dsDNA prophage into a single prediction (NgoΦ2 with NgoΦ6; NgoΦ3 with NgoΦ9) (Fig. S1A). This difficulty may be due in part to the characteristic low sequence identity between filamentous phages and their small sizes (PhiSpy imposes a cutoff for the minimum number of genes in a prophage region to be called a prophage [22, 50]).

Actively replicating prophages result in high prophage-to-host read coverage ratios (54). We used hafeZ (55) and PropagAtE (54) to investigate whether the predicted prophages may be active in a subset of genomes for which Illumina reads are available (see Materials and Methods; Table S1, tab C). No active prophages were identified by PropagAtE, and only seven by hafeZ (two of which overlap with Seeker predictions), suggesting that most prophages in the subset of genomes examined do not produce virions.

To exclude identical predictions in subsequent analyses, we performed dereplication at 95% length aligned (see Materials and Methods; Table S2, tab B), resulting in 1,302 unique predictions. No phages identified in different bacterial species were found to be similar at ≥95% length aligned (Table S2, tab B). The distribution of lengths of dereplicated prophages predicted by each tool is shown in Fig. S2.

10.1128/msystems.00083-22.2 FIG S2 The distribution of predicted prophage lengths for each virus prediction tool. (A to C) The length in kb is shown for dereplicated PhiSpy (A), VirSorter2 (B), and (C) Seeker predictions. Information about each prediction is provided in Table S2, tab A and dereplication of predictions in Table S2, tab B. Download FIG S2, TIF file, 2.8 MB. Copyright © 2022 Orazi et al. 2022 Orazi et al. https://creativecommons.org/licenses/by/4.0/ This content is distributed under the terms of the Creative Commons Attribution 4.0 International license.

Subsequently, we present analyses on predictions made by all three tools (Table S2, tab A) and 13 known *Neisseria* phages (Table S1, tab B). For the sake of clarity, analyses of PhiSpy predictions are presented in the main text, while analyses of VirSorter2 and Seeker predictions are included in the supplemental material. We focus on a single tool to avoid the issue of reconciling overlapping predictions between tools and selected PhiSpy because it more accurately predicted the boundaries of known *Neisseria* prophages (Fig. S1).

### Few predictions are similar to known *Neisseria* plasmids and the gonococcal genetic island.

In this study, we used prediction tools that search for viruses. However, because VirSorter2 has been reported to have difficulty distinguishing plasmids from viral sequences (51, 56, 57), we wanted to address the possibility that predictions from any tool may resemble other types of *Neisseria* MGEs.

Specifically, we compared our predictions to known *Neisseria* plasmids and the gonococcal genetic island (GGI). To perform this analysis, we performed hierarchical clustering based on percent length aligned of dereplicated predictions and nucleotide sequences of plasmids and the GGI obtained from GenBank (47, 48) (Table S1, tab D).

Only 14 unique predictions cluster with *Neisseria* plasmids and the GGI based on nucleotide sequence (Fig. S3, Data set S1). Of these 14 predictions, 2 (both predicted by VirSorter2) cluster with known *Neisseria* plasmids (Fig. S3A), and 12 predictions (all predicted by Seeker) cluster with the GGI (Fig. S3B).

10.1128/msystems.00083-22.3 FIG S3 Few predictions cluster with known *Neisseria* plasmids or genetic islands based on percent length aligned nucleotide sequences. (A and B) Dendrograms representing clustering of dereplicated predictions from all tools with known *Neisseria* plasmids (A) and the gonococcal genetic island (GGI) (B). Average-linkage hierarchical clustering was performed using SciPy based on percent length aligned nucleotide sequence, and the resulting dendrograms were visualized with iTOL. In panel A, individual clusters are highlighted by alternating light and dark gray clade shading. Beneath the dendrogram, color strips indicate the *Neisseria* species in which the MGE was identified (top strip) and whether the MGE is a known plasmid/GGI or a predicted prophage (bottom strip). Only predictions that cluster with known *Neisseria* plasmids or the GGI are included in the dendrograms. Information about plasmids and the GGI are provided in Table S1, tab D. Hierarchical clustering memberships of each indicated cluster are presented in Data set S1, tab 2. Download FIG S3, TIF file, 1.6 MB. Copyright © 2022 Orazi et al. 2022 Orazi et al. https://creativecommons.org/licenses/by/4.0/ This content is distributed under the terms of the Creative Commons Attribution 4.0 International license.

Because our study focuses on phages, we excluded these 14 predictions from our subsequent analyses. The results described above indicate that the majority of predictions in this study are dissimilar to known *Neisseria* plasmids and the GGI.

### Comparing predicted prophages to known phages using gene-sharing networks.

**FIG 2:**
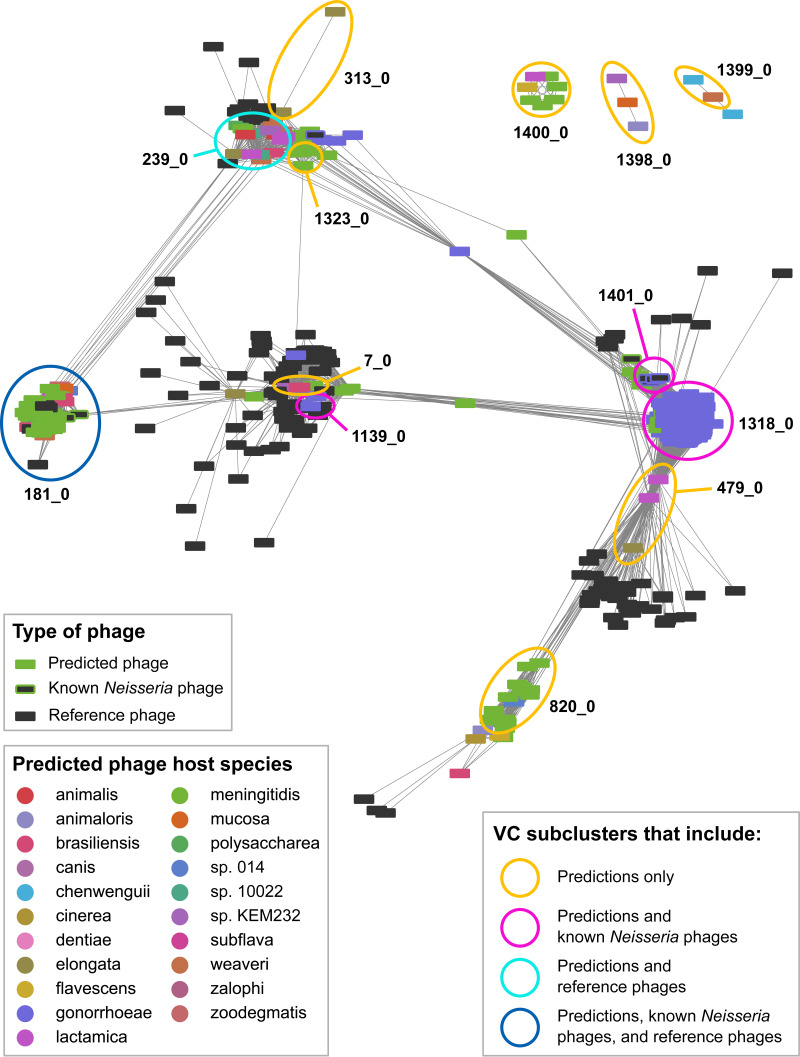
vConTACT clustering of PhiSpy predictions, known *Neisseria* phages, and phages that infect other bacterial taxa. vConTACT v2.0-generated network of dereplicated PhiSpy predictions and known phages visualized with Cytoscape using an edge-weighted spring-embedded algorithm. Nodes represent predicted prophages (color corresponding to the *Neisseria* species in which the prophage was identified), known *Neisseria* phages (dark gray outlined in the color corresponding to the bacterial host species), or phages that infect other bacterial taxa (i.e., reference viruses; dark gray without outline). Edges represent the vConTACT-generated similarity score between each pair of viruses (only similarity scores of ≥1 are included in the network). Highly similar viruses are positioned close together. Only reference viruses that are connected to ≥1 predicted prophage are included in the network. Information about vConTACT subclusters is included in Data set S1, tab 3, and similarity scores (edge weights) in tab 4.

Classifying phages is challenging due to their high genomic diversity, extensive mosaicism, and lack of universally shared genes (58–61). Therefore, gene-sharing networks are commonly used to compare novel phages to previously identified phages (38, 39, 61–65). Here, we used vConTACT v.2.0 (64, 66) to assess whether the prophages we predicted are similar to known *Neisseria* phages (Table S1, tab B) or phages that infect other bacterial taxa (i.e., reference viruses; see Materials and Methods). vConTACT generates a similarity score between each pair of viruses based on the protein clusters they share. If two viruses are significantly similar to one another (i.e., the pair has a score of ≥1), then they are connected by an edge. Groups of viruses that are highly similar are placed within the same subcluster and are likely members of the same viral genus (64). We used vConTACT to separately analyze dereplicated predictions from each tool, resulting in three distinct networks (Fig. 2, Fig. S4A and B).

10.1128/msystems.00083-22.4 FIG S4 vConTACT clustering of known *Neisseria* phages, phages that infect other bacterial taxa, and either VirSorter2 or Seeker predictions. (A and B) vConTACT v2.0-generated networks of dereplicated VirSorter2 (A) or Seeker (B) predictions with known *Neisseria* phages and viruses that infect other bacterial taxa (i.e., reference viruses). Networks were visualized with Cytoscape using an edge-weighted spring-embedded algorithm. Nodes represent predicted prophages (color corresponding to the *Neisseria* species in which prophages were identified), known *Neisseria* phages (dark gray outlined in the color corresponding to the bacterial host species), or reference viruses (dark gray without outline). Edges show the vConTACT-generated similarity score between each pair of viruses (only similarity scores of ≥1 are included in the network). Highly similar viruses are positioned close together. Only reference viruses that connect to ≥1 predicted prophage are included in the network. Information about vConTACT subclusters is included in Data set S1, tab 3, and similarity scores (edge weights) in tabs 4 to 6. Download FIG S4, TIF file, 2.3 MB. Copyright © 2022 Orazi et al. 2022 Orazi et al. https://creativecommons.org/licenses/by/4.0/ This content is distributed under the terms of the Creative Commons Attribution 4.0 International license.

First, we examined whether PhiSpy predictions are significantly similar to known *Neisseria* phages (i.e., connected by an edge in the network). While 83% of PhiSpy predictions (229/277) are connected to known *Neisseria* phages, only 52% of PhiSpy predictions (144/277) cluster with known *Neisseria* phages (Table S2, tab C). These 144 predictions belong to the following four subclusters: 181_0 (dark blue circle) and 1139_0, 1401_0, and 1318_0 (pink circles, Fig. 2). Thus, only half of PhiSpy predictions are likely members of the same viral genus as known *Neisseria* phages.

Next, we compared PhiSpy predictions to viruses that infect bacterial taxa other than *Neisseria* (i.e., reference viruses). We found that 86% of PhiSpy predictions (239/277) are significantly connected to reference viruses (Table S2, tab C). However, only 15% of PhiSpy predictions (42/277) cluster with reference viruses (Table S2, tab C). These 42 predictions belong to either subcluster 181_0 (dark blue circle) or 239_0 (light blue circle, Fig. 2); below, we explore these 2 subclusters that contain both PhiSpy predictions and reference viruses.

**FIG 3:**
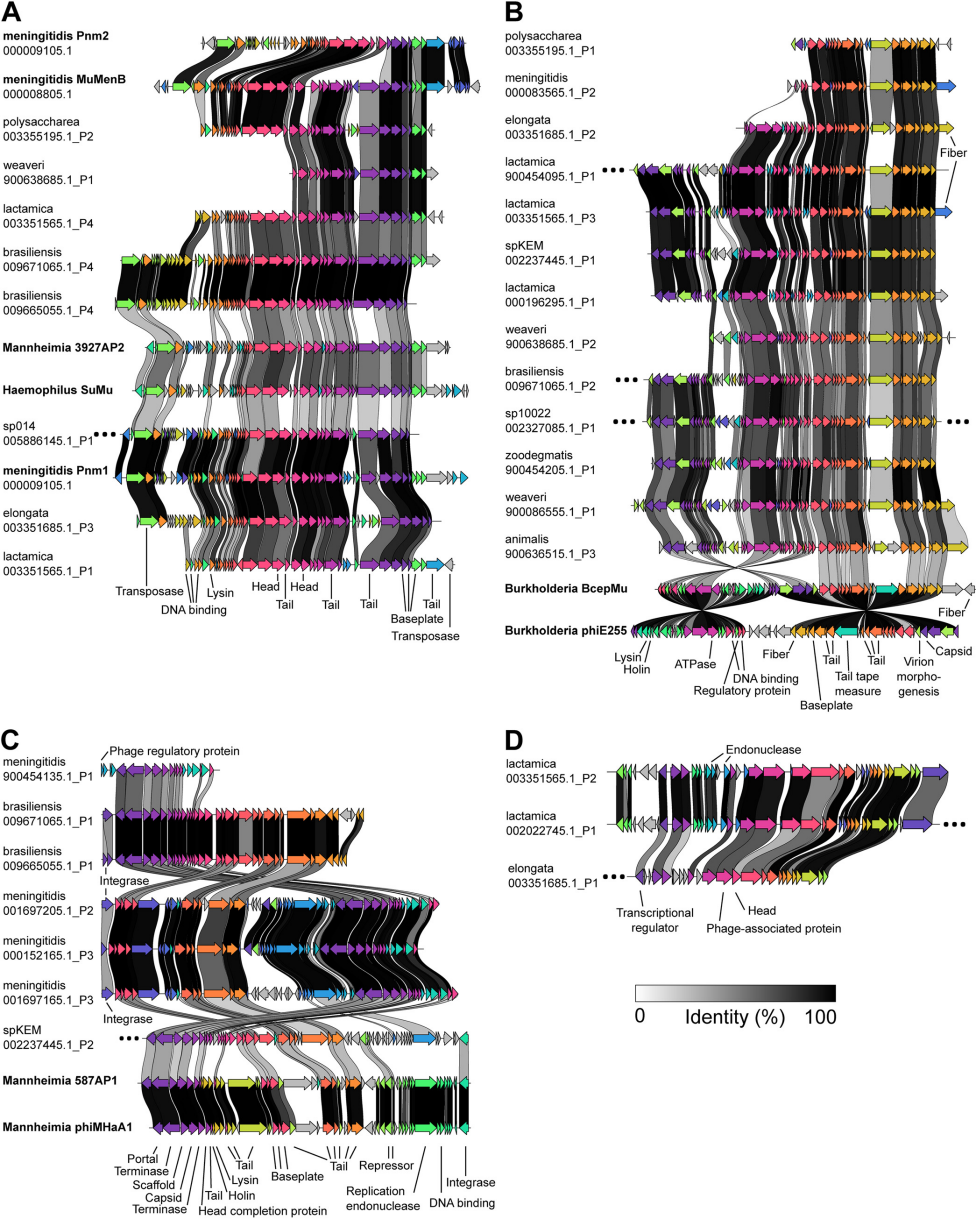
Genes shared between PhiSpy predictions, known *Neisseria* phages, and phages that infect other bacterial taxa. (A to D) Clinker-generated visualizations showing genes that are shared between members of the following vConTACT subclusters: 181_0 (A), 239_0 (B), 7_0 (C), and 479_0 (D). The location of each subcluster within the vConTACT-generated network is shown in Fig. 2. Known *Neisseria* phages and phages that infect other bacterial taxa (i.e., reference phages) are indicated in bold. Arrows with the same color indicate genes that are similar between phages; connections between arrows indicate amino acid sequence identity as described in the key. Gray arrows indicate genes that are not shared between phages; ellipses indicate that >2 unshared genes are present at either end of a prophage genome. Shared genes are annotated with predicted functions of encoded proteins (except for hypothetical proteins). In panel A, several late phage genes are present/absent between *Burkholderia* phages and predicted *Neisseria* phages (gene order in phiE255 from left to right: lysin, holin, tail tape measure, fiber). Panel C also includes two reference viruses that do not belong to subcluster 7_0, *Mannheimia* phages 587AP1 and phiMHaA1. Information about vConTACT subclusters is included in Data set S1, tab 3.

Subcluster 181_0 includes Mu-like phages that infect N. meningitidis (Pnm1-2, MuMenB), Mannheimia haemolytica (3927AP2), and Haemophilus parasuis (SuMu, shown in bold, Fig. 3A). Previously, Pnm1-2 and MuMenB were found to resemble a Mu-like phage that infects Haemophilus influenzae (24). There is a high degree of synteny between members of 181_0, and the proteins shared between known *Neisseria* prophages and the predictions in this subcluster have >50% sequence identity (Fig. 3A).

Similarly, subcluster 239_0 contains two Mu-like phages that infect Burkholderia cenocepacia (BcepMu) and Burkholderia thailandensis (phiE255, Fig. 3B). Except for several late phage genes, most predicted proteins are shared between the *Burkholderia* phages and *Neisseria* predictions (at ∼30 to 50% sequence identity, Fig. 3B). Together, these results suggest that several *Neisseria* species may be infected by phages similar to those that infect Haemophilus, *Mannheimia*, and *Burkholderia*—microbes that *Neisseria* species may encounter within the respiratory tracts of humans and/or animals.

We also explored subcluster 7_0 (orange circle, Fig. 2). Although it does not include any reference viruses, members of 7_0 share many genes with reference viruses (many surrounding dark gray nodes, Fig. 2). In particular, a *Neisseria* sp. KEM 232 prediction belonging to 7_0 shares 48% of predicted proteins (29/60) with other members of 7_0 (Fig. 3C) and also shares 30% of proteins (18/60) with two *Mannheimia* P2-like phages that do not belong to this subcluster (587AP1 and phiMHaA1, Fig. 3C).

While the majority of PhiSpy predictions do not cluster with reference phages (Table S2, tab C), many predictions were found to have a low degree of similarity to different reference phages (as indicated by the low similarity scores between predictions and reference viruses, Fig. S5). For example, the N. lactamica and N. elongata predictions belonging to subcluster 479_0 (orange circle, Fig. 2; Fig. 3D) each have a low degree of similarity to ∼20 to 50 different reference viruses (Fig. 2). Therefore, these findings suggest that many PhiSpy predictions are distantly related to multiple viruses that infect other bacterial taxa.

10.1128/msystems.00083-22.5 FIG S5 PhiSpy and VirSorter2 predictions are more similar to known viruses than to Seeker predictions. vConTACT v2.0-generated similarity scores between each predicted prophage and its most similar known virus in the network. Known viruses include both known *Neisseria* phages and reference phages. Similarity scores are shown for each virus pair and grouped by the tool that predicted the prophage (PhiSpy, VirSorter2, or Seeker). Distributions of similarity scores were compared between each tool using the Mann-Whitney U test implemented in SciPy. Similarity scores (edge weights) for each network are provided in Data set S1, tabs 4 to 6. Download FIG S5, TIF file, 1.5 MB. Copyright © 2022 Orazi et al. 2022 Orazi et al. https://creativecommons.org/licenses/by/4.0/ This content is distributed under the terms of the Creative Commons Attribution 4.0 International license.

Finally, we found differences in how similar predictions from each tool are to known *Neisseria* phages and reference phages (together referred to as “known phages”). Specifically, (i) a smaller proportion of Seeker predictions are connected to known phages compared to PhiSpy and VirSorter2 predictions (Table S2, tab C), (ii) the degree to which Seeker predictions are similar to known phages is significantly lower than those of the other tools, as indicated by lower similarity scores (Fig. S5), and (iii) zero Seeker predictions cluster with known phages (Table S2, tab C). Thus, Seeker predictions may represent novel phages, other MGEs, or alternatively, regions of the chromosome that were incorrectly called.

### Highly similar predicted prophages are found in distantly related *Neisseria* species.

**FIG 4:**
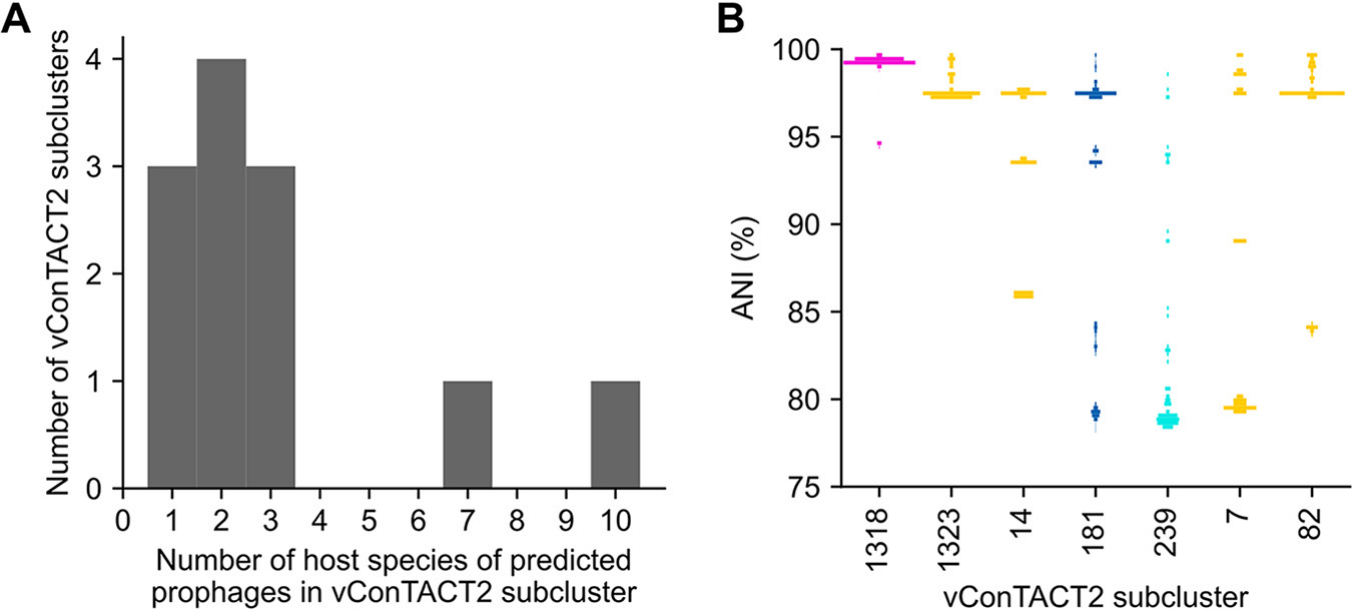
Several vConTACT subclusters include PhiSpy predictions from different *Neisseria* host species. (A) For each vConTACT v2.0 subcluster, the *Neisseria* host species of each PhiSpy prediction was determined. The histogram shows how many subclusters (*y* axis values) include the indicated numbers of unique host species (*x* axis values). (B) The percent average nucleotide identity (ANI) between the *Neisseria* genomes in which PhiSpy predictions were identified. Only vConTACT subclusters that include 5 or more predicted prophages are shown (“_0” was omitted from the end of subcluster names). The distribution of ANI values is represented as a histogram where the width of bars at a given ANI corresponds to the proportion of genome pairs with that ANI. The color of each subcluster indicates the types of predictions that belong to that subcluster (orange, predictions only; pink, predictions and known *Neisseria* phages; light blue, predictions and reference phages; dark blue, predictions, known *Neisseria* phages, and reference phages). Information about vConTACT subclusters is included in Data set S1, tab 3.

Next, we explored whether different *Neisseria* species may be infected by highly similar phages by examining whether any vConTACT subclusters include PhiSpy predictions found in genomes of different *Neisseria* species (Fig. 4A).

Out of the 12 subclusters that include PhiSpy predictions, 9 contain predictions identified in different *Neisseria* species (Fig. 4A). Strikingly, subclusters 181_0 and 239_0 (Fig. 3A and B) include predictions found in 7 and 10 different *Neisseria* species, respectively (Fig. 4A).

We also investigated whether the bacterial species (in which the predictions were identified) are closely or distantly related to each other. For every subcluster that includes ≥5 predictions, we calculated the pairwise average nucleotide identity (ANI) between each bacterial genome in which the predicted prophages were identified.

Every subcluster we examined includes multiple phages found in the same species, as shown by an ANI of >95% (Fig. 4B). Additionally, four subclusters include predictions found in closely related species (ANI, 90 to 95%).

Finally, five subclusters contain predictions found in more distantly related species (ANI, <90%; Fig. 4B), including three subclusters with an ANI of ∼80% (181_0, 239_0, 7_0; Fig. 4B) that were highlighted above (Fig. 3). Therefore, these results suggest that even distantly related *Neisseria* species may be infected by closely related phages.

### Identification of CRISPR arrays and spacer matches in *Neisseria* genomes.

Here, we surveyed a larger set of 2,619 *Neisseria* genomes (see Materials and Methods, Table S1, tab E) for the presence of CRISPR arrays (Data set S1). Consistent with previous findings (43–45), we identified type II-C CRISPR arrays in 45% of N. meningitidis genomes (862/1,894) and no CRISPR arrays in N. gonorrhoeae genomes (0/630).

In addition, we identified CRISPR arrays in ≥1 genome of every commensal species included in this study. Repeat sequences in arrays of commensal species are associated with seven different CRISPR subtypes (I-A, I-C, I-F, II-C, III-A, III-B, III-D). In total, we found 3,676 unique CRISPR spacers (Data set S1).

Next, we used BLASTn to search for matches between CRISPR spacers and sequences in *Neisseria* genomes. We only kept matches that had 100% identity over the entire length of the spacer (i.e., 0 mismatches), and we looked for both intra- and interspecies matches.

We found that 22% of spacers (820/3,676) target sequences in the smaller set of high-quality *Neisseria* genomes. Out of these targeting spacers, 66% (539/820) match known or predicted prophages. Previously, Zhang et al. identified five self-targeting spacers in six N. meningitidis genomes (44). Here, we found that 52% of CRISPR-positive high-quality N. meningitidis genomes (23/44) encode self-targeting spacers.

### Examining the locations of CRISPR matches in *Neisseria* genomes.

We next examined the genomic locations of spacer matches. In addition to providing defense against MGEs, the type II-C CRISPR system of N. meningitidis has been proposed to play a role in limiting natural transformation (44, 45). If CRISPR systems restrict transformation, we would expect to see targeting evenly distributed along the length of the bacterial chromosome with no obvious enrichment of targeting in any location. If, however, prophages are targeted by CRISPR immunity, we would expect that matches would be enriched in prophages.

**FIG 5:**
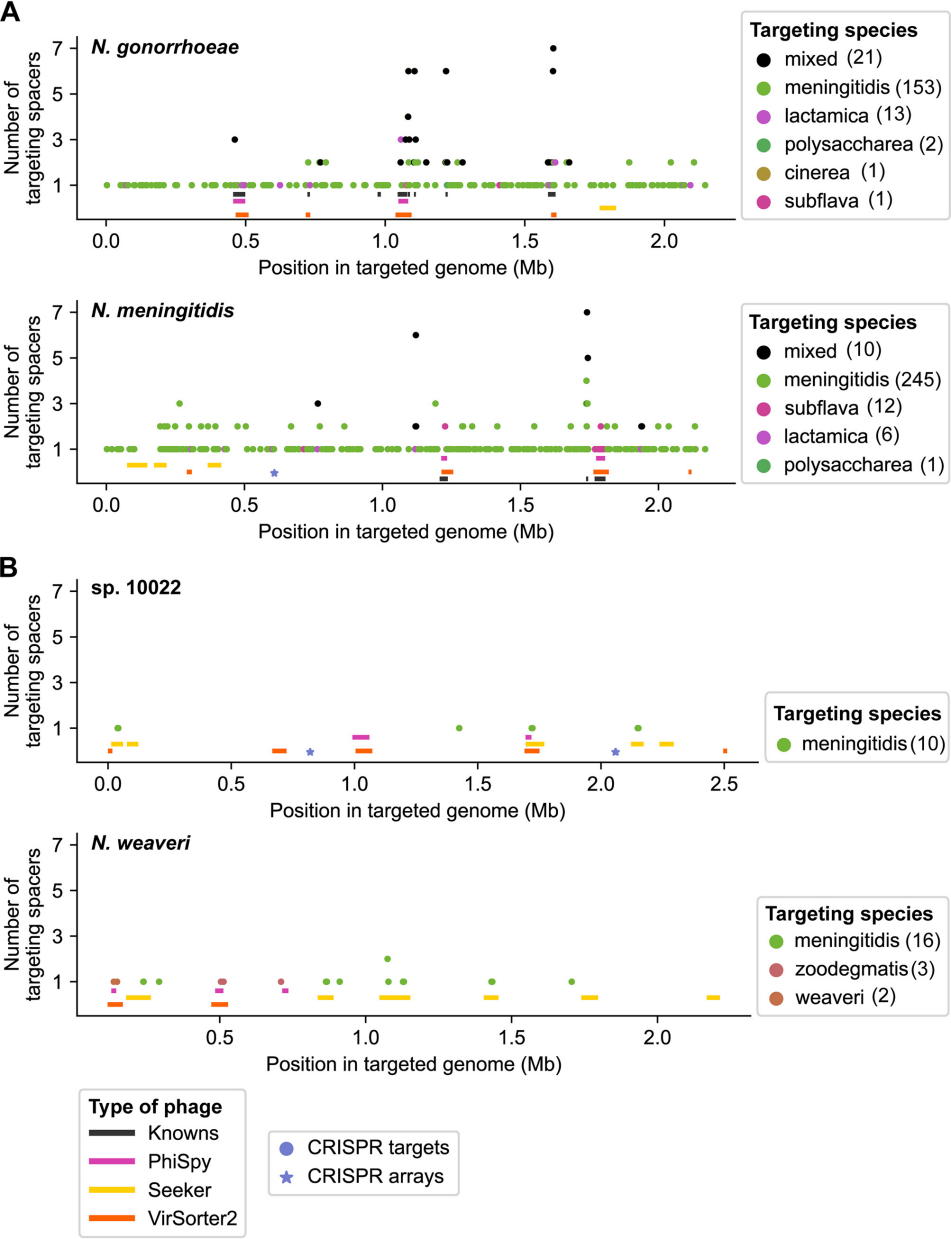
Locations of matches between CRISPR spacers and *Neisseria* genomes. (A and B) Genomic locations of matches between *Neisseria* CRISPR spacers and bacterial genomes (at 100% identity over the entire spacer length). Each plot shows a genome of a different species—N. gonorrhoeae (GenBank assembly version no. GCA_000006845.1) and N. meningitidis (GCA_000009105.1) (A); sp. 10022 (GCA_002327085.1) and *N. weaveri* (GCA_900638685.1) (B). Each data point (circle) represents the number of spacers that match each position (1-kb bin) in the bacterial genome. The color of the circle corresponds to the species encoding the spacer (as indicated in the key, with mixed indicating >1 unique species). In parentheses is the number of spacers encoded by each species that match the genome. CRISPR arrays are denoted by stars, and prophages are represented by rectangles (the type of prophage is indicated in the key). Information about CRISPR targeting of each genome is provided in Data set S1, tab 10. Locations of CRISPR arrays are included in tab 7. Locations of known and predicted prophages are found in Table S1, tab B and Table S2, tab A, respectively.

Figure 5 shows the genomic locations of spacer matches in two genomes that encode CRISPR arrays (N. meningitidis, sp. 10022) and two that do not (N. gonorrhoeae, N. weaveri). In N. gonorrhoeae and N. meningitidis genomes, we observe a low level of targeting across the length of the genome (Fig. 5A), consistent with inhibiting transformation. There are also regions of high targeting; in N. gonorrhoeae, peaks correspond to the location of several known prophages, whereas peaks in the N. meningitidis genome may correspond to as yet unidentified MGEs (Fig. 5A).

Matches in sp. 10022 and *N. weaveri* genomes primarily correspond to predicted prophages (Fig. 5B). Overall, many fewer spacers appear to target sp. 10022 and *N. weaveri* genomes; this is likely due at least in part to the few available genomes of commensal species (Table S1, tab F), leading to a small pool of targeting spacers. Thus, our ability to make comparisons of targeting between N. meningitidis and commensals is limited.

Finally, we examined which species encode the targeting spacers (color of each circle, Fig. 5). Previously, N. meningitidis spacers were reported to match protospacers in N. gonorrhoeae genomes (44); here, we observe that N. meningitidis is largely responsible for the low-level targeting of the N. gonorrhoeae and N. meningitidis genomes in Fig. 5A. In contrast, prophages in these four genomes are matched by spacers from N. meningitidis or (an)other species (Fig. 5). In subsequent analyses, we quantify CRISPR targeting of each prophage and bacterial genome and further investigate interspecies targeting.

### Comparing CRISPR targeting of each predicted prophage to backbone targeting.

Above, we observed CRISPR targeting along the entire length of the chromosome in N. gonorrhoeae and N. meningitidis (Fig. 5A). To distinguish whether predicted prophages are preferentially targeted, it is necessary to compare the level of targeting of predicted prophages to the background level across the rest of the genome. Therefore, we quantified the density of CRISPR targeting of every predicted prophage and the rest of the bacterial genome in which it was identified (i.e., the backbone).

We define prophage targeting density as the number of CRISPR matches in the prophage divided by the prophage length. Backbone targeting density is obtained by dividing backbone targeting (the number of CRISPR matches in a bacterial genome excluding targets in all known or predicted prophages and CRISPR arrays) by the length of the backbone (length of the entire bacterial genome minus the combined lengths of the prophages identified in that genome).

**FIG 6:**
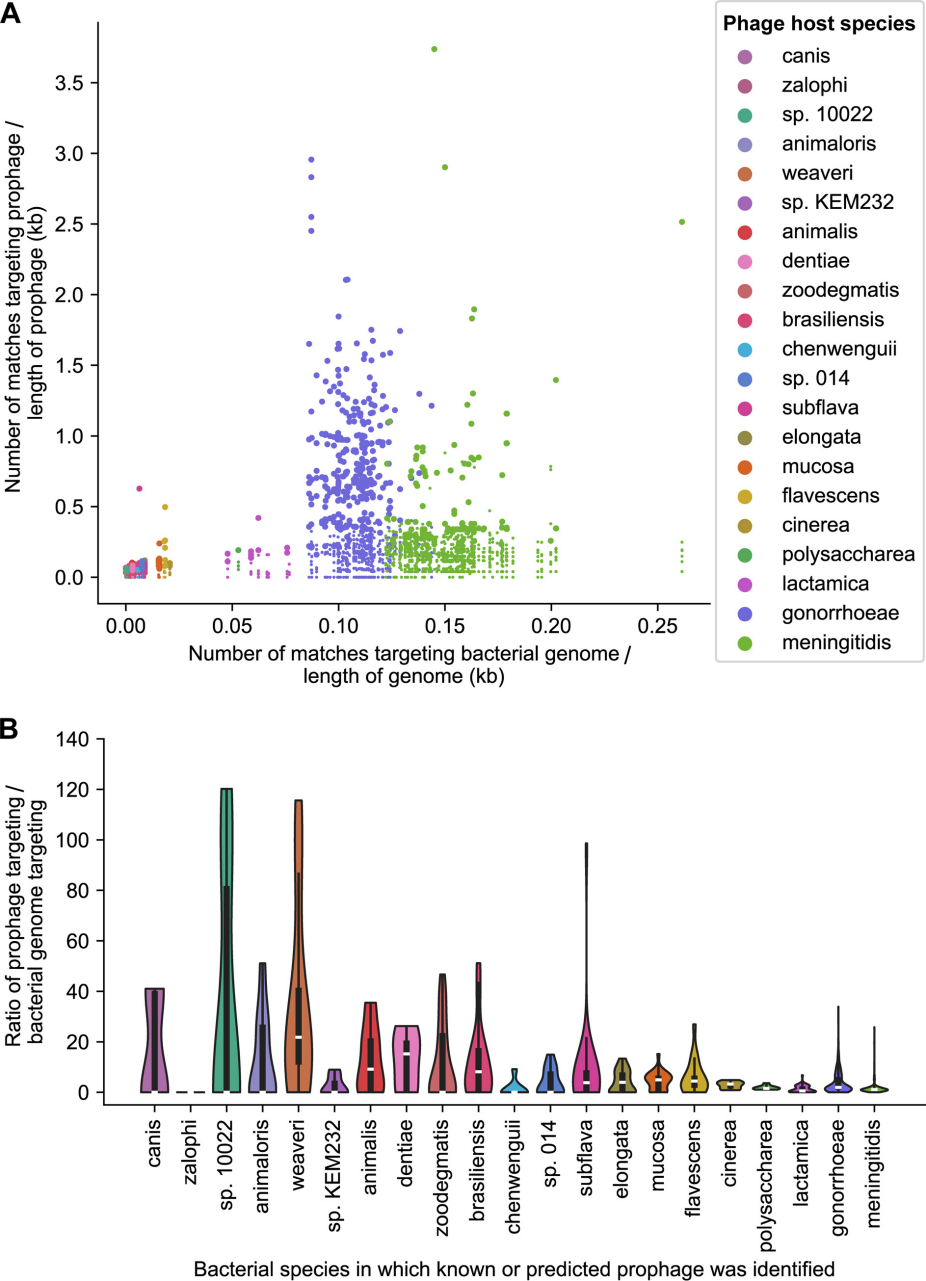
Comparing CRISPR targeting densities of prophages and bacterial genome backbones. (A) Matches were identified between CRISPR spacers and bacterial genomes in the smaller genome data set (at 100% identity over the entire spacer length). Then, targeting densities were determined for every bacterial genome, known *Neisseria* prophage, and dereplicated prophage predicted by PhiSpy, VirSorter2, and Seeker. For each prophage, the density of matches in the prophage was compared to the density of matches in the rest of genome in which the prophage was identified (i.e., the backbone). *y* coordinates represent the number of matches in each prophage divided by the length of the prophage. *x* coordinates represent the number of matches in the backbone (excluding spacers targeting all known or predicted prophages in that genome) divided by the length of the backbone (excluding the length of all known or predicted prophages in that genome). Each data point represents a known *Neisseria* prophage or a predicted prophage. The color of the circle corresponds to the species in which the prophage was identified. Larger circles indicate prophages that have significantly higher targeting densities compared to the backbone (statistical testing is described in Materials and Methods). (B) Violin plot showing the distribution of prophage/backbone CRISPR targeting ratios from panel A grouped according to the species in which the prophage was identified. CRISPR targeting densities and ratios are provided in Data set S1, tab 10, and significantly targeted prophages are listed in tab 11.

First, we compared the targeting density of each prophage to the targeting density of the backbone and determined which prophages are significantly more highly targeted than the backbone (Fig. 6A). We then compared ratios of prophage/backbone targeting between different *Neisseria* species (Fig. 6B).

Although many N. gonorrhoeae and N. meningitidis prophages have high targeting densities (Fig. 6A), the high degree of backbone targeting of these genomes results in mostly low targeting ratios (Fig. 6B). For example, even though all 13 known *Neisseria* prophages are matched by spacers, only 5 of them (MDAΦ and NgoΦ6 to -9) are significantly more highly targeted than the backbone (Data set S1).

In multiple commensal species, the ratio of prophage/backbone targeting is very high (Fig. 6B), in many cases due to little or no backbone targeting (Fig. 6A). Low levels of backbone targeting could be due to several, nonmutually exclusive reasons: (i) primarily, the small number of commensal spacers sampled in this study resulting in lower apparent targeting, (ii) infrequent encounters between certain species (e.g., *N. weaveri* is an opportunistic pathogen rather than a resident of the human mucosa) (67), or (iii) species-specific barriers to transformation, including differences in DNA uptake sequences (68) and in whether CRISPR systems target chromosomal DNA (44, 45).

Overall, 20% of dereplicated prophages predicted in this study (259/1,306) have a significantly higher targeting density than the backbone (Table S2, tab C, Data set S1). Furthermore, the majority of significantly targeted predictions (74%; 191/259) do not cluster with known *Neisseria* phages, plasmids, or the GGI (Table S2, tab C). These 191 predictions belong to 30 different vConTACT subclusters (Data set S1), including 3 PhiSpy subclusters highlighted above (239_0, 7_0, 479_0; Fig. 3). Therefore, these 191 predictions represent likely candidates for novel *Neisseria* prophages.

### Interspecies CRISPR targeting is widespread among *Neisseria* species.

We observed a high degree of interspecies targeting in our data set. Out of 539 spacers that target known or predicted prophages, 288 spacers are involved in interspecies targeting of prophages. Furthermore, 186 spacers only target prophages found in another species (and not prophages found in genomes of their own species).

**FIG 7:**
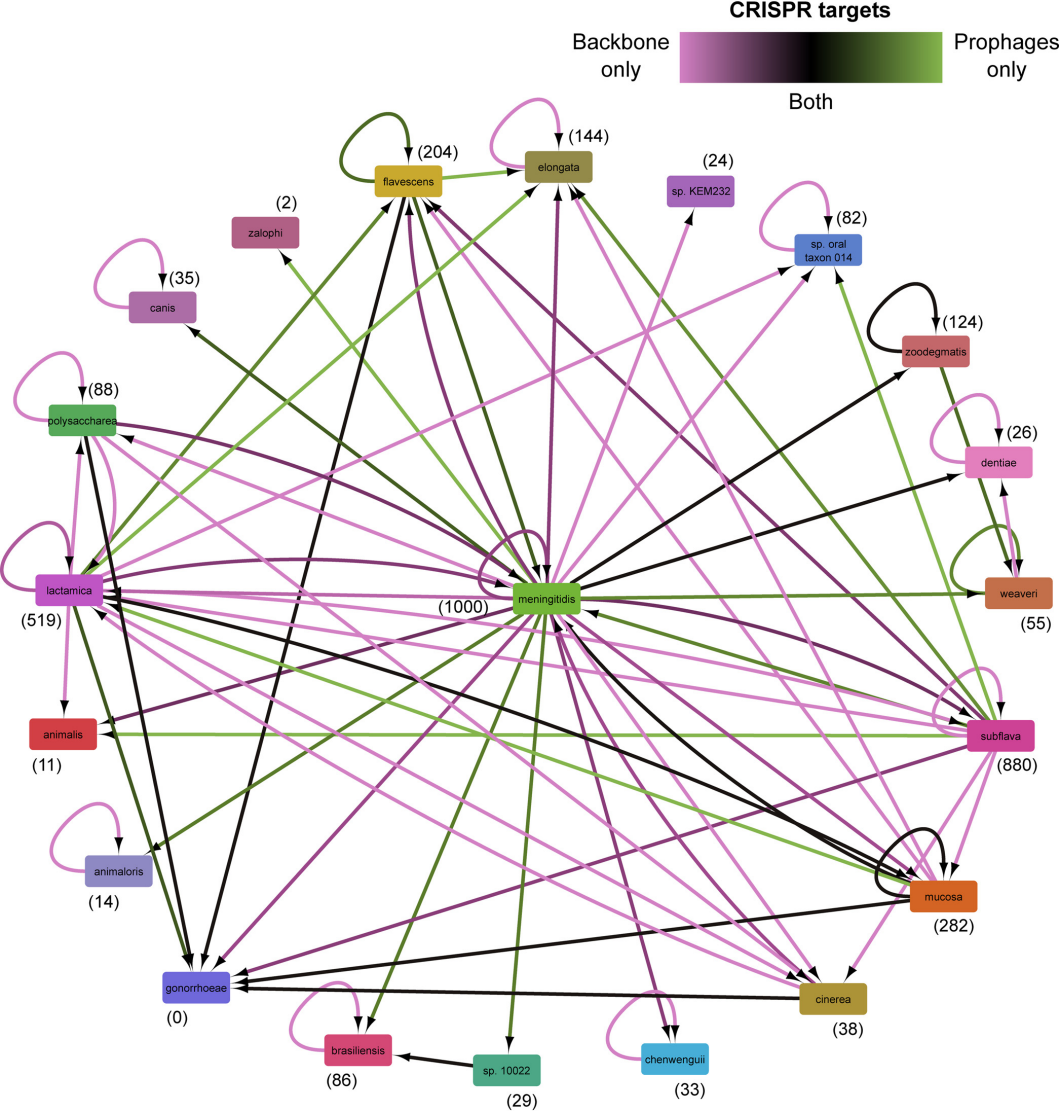
Interspecies CRISPR targeting of *Neisseria* prophages and bacterial genome backbones. Network representing intra- and interspecies CRISPR targeting of prophages and the rest of the genome in which the prophage was identified (i.e., the backbone) visualized using Cytoscape. The prophages included in this analysis are known *Neisseria* prophages and dereplicated prophages predicted by PhiSpy, VirSorter2, and Seeker that are targeted significantly more than the backbone and do not cluster with *Neisseria* plasmids. Each node represents a bacterial species, and the adjacent number in parentheses indicates the total number of spacers encoded by that species. Nodes are connected by an edge if CRISPR spacers encoded by one species target another species. The direction of CRISPR targeting is indicated using an arrow that points to the species being targeted. Edge color indicates the relative number of spacers targeting prophages compared to the targeting of the backbone (excluding CRISPR arrays or sequence contained in any known or predicted prophages—not only predictions that are significantly targeted). Information about CRISPR spacers and targeting is provided in Data set S1, tabs 9 to 10.

We further explored interspecies CRISPR targeting using a network to represent targeting relationships between *Neisseria* species (Fig. 7). This network includes targeting of known prophages, predictions from all three tools, and backbone sequences (defined above). To increase the likelihood of examining phages (instead of chromosomal or plasmid sequences), we only included dereplicated predictions that have significantly higher targeting densities than those of the backbone (i.e., significantly targeted predictions) and that do not cluster with *Neisseria* plasmids.

The network is highly interconnected; all 21 *Neisseria* species included in this study are connected to ≥1 other species in the network, and 16 species are connected to ≥2 others (Fig. 7). Interestingly, N. meningitidis spacers match prophage and backbone sequences of 17 and 20 different species, respectively, and N. gonorrhoeae and N. meningitidis are each targeted by 7 *Neisseria* species.

Moreover, there are differences in the type of sequences targeted (edge color, Fig. 7). N. meningitidis spacers predominantly match backbone genome sequences of N. meningitidis and several other species (many pink arrows pointing from N. meningitidis). In contrast, N. subflava and N. lactamica spacers primarily target prophages of other species (mostly green arrows pointing from *N. subflava* and N. lactamica).

While these results suggest that interspecies targeting of *Neisseria* sequences is widespread, an alternative explanation is that spacers were exchanged between species. However, out of 3,676 total spacers, only 2 identical spacers were present in genomes from different species (Data set S1). Taken together, the findings described above may indicate that interspecies CRISPR targeting is common between *Neisseria* species.

Finally, we investigated whether *Neisseria* prophages may be targeted by other bacterial taxa using CRISPRopenDB and its database of 11 million spacers (69). Four predictions identified in N. animalis are matched by the same, single spacer from Eikenella corrodens (another member of the Neisseriaceae), while an *N. elongata* prediction is matched by one spacer from Aggregatibacter aphrophilus (Data set S1).

### Many known and predicted *Neisseria* prophages have additional inferred host species.

Elucidating the host range of phages is critical for understanding how they influence their microbial hosts, including their role in mobilizing DNA (15). Since CRISPR targeting data are frequently used to predict the bacterial hosts of phages (38–42), we took advantage of the extensive interspecies CRISPR targeting observed above to perform a similar analysis.

**FIG 8:**
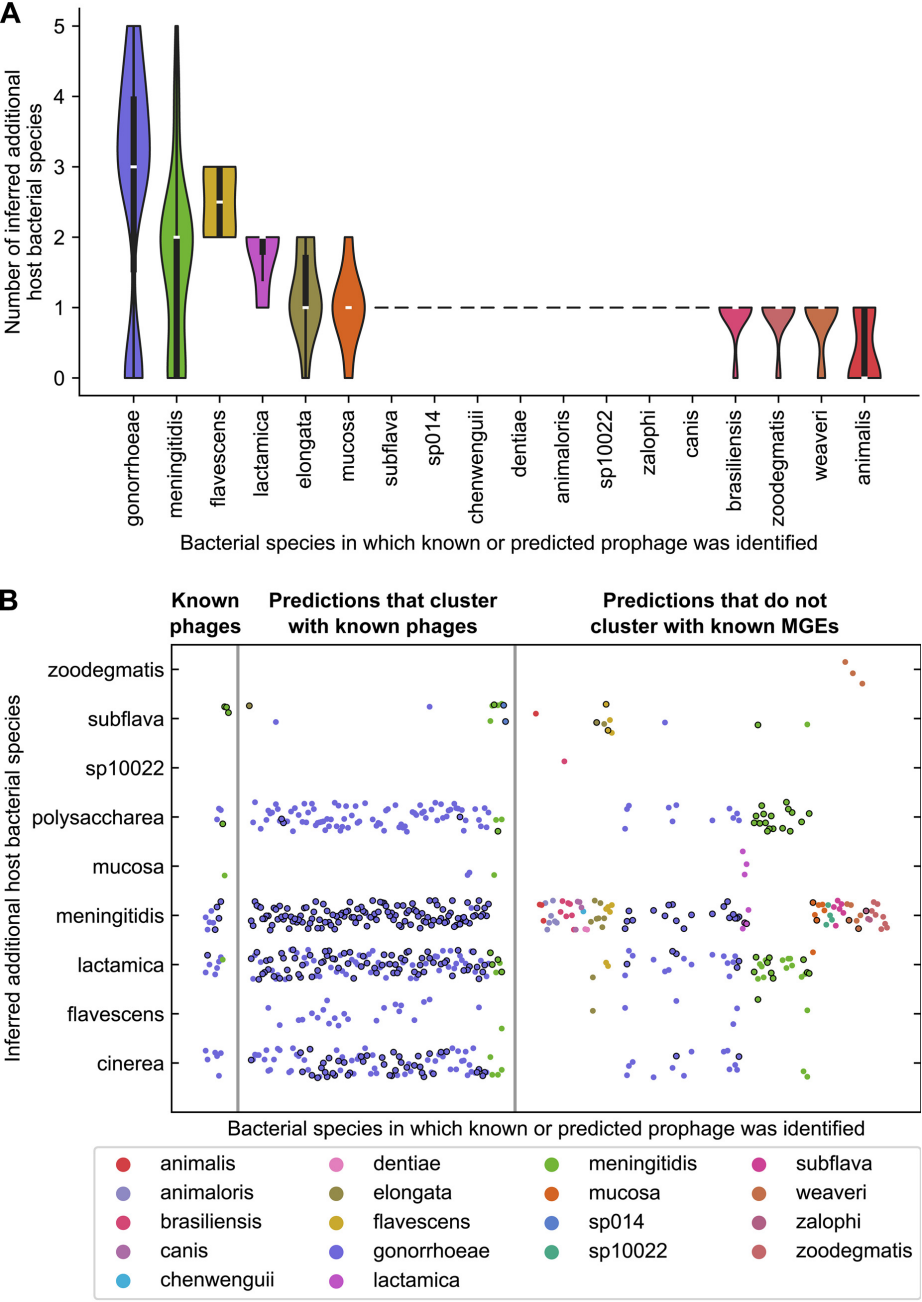
Inference of additional host species of *Neisseria* phages using interspecies CRISPR targeting data. (A) Violin plot showing the distribution of the number of inferred additional host species of known *Neisseria* phages and dereplicated predictions made by PhiSpy, VirSorter2, and Seeker that have a significantly higher CRISPR targeting density than the backbone. Data are grouped according to the species in which the prediction was identified. Interspecies matches between CRISPR spacers and prophages were used to infer phage host species in addition to the species in which the phage was identified. (B) Data from panel A shown for each prophage that has ≥1 inferred additional host species (the species names are along the *y* axis). Each circle represents a known or predicted prophage, and the color of the circle corresponds to the species in which the prophage was identified (as indicated in the key). Circles with a black outline indicate that >1 spacer encoded by that species matches that prophage, while circles without an outline represent a single spacer match. Phages are categorized as follows: known *Neisseria* phages, predicted prophages that cluster with known *Neisseria* prophages, and predicted prophages that do not cluster with known *Neisseria* MGEs (phages, plasmids, or the gonococcal genetic island).

Specifically, we inferred additional hosts of known *Neisseria* prophages and significantly targeted predictions (defined above) from PhiSpy, VirSorter2, and Seeker (see Materials and Methods). Our findings suggest that 254/259 significantly targeted predictions and all 13 known *Neisseria* phages have ≥1 additional host species (Fig. 8A, Table S2, tab D) and that prophages are shared among a variety of *Neisseria* species (Fig. 8B).

N. gonorrhoeae predictions, in particular, have broad inferred host ranges (Fig. 8A). All (145/145) significantly targeted N. gonorrhoeae predictions have ≥2 inferred additional host species, and 72/145 have ≥4 additional host species (Table S2, tab D). N. gonorrhoeae phages (dark purple dots, Fig. 8B) are shared primarily with three closely related species, N. meningitidis, N. polysaccharea, and N. lactamica (median ANI between each species and N. gonorrhoeae, 93 to 95%; Fig. 1), and also with N. cinerea, which is less closely related (median ANI, 90%).

Furthermore, N. meningitidis is predicted to share phages with many species, especially predictions that do not cluster with known *Neisseria* MGEs (many differently colored dots in the N. meningitidis row, Fig. 8B). Among these species, seven (N. animaloris, N. canis, N. dentiae sp. 10022, *N. weaveri*, N. zalophi, N. zoodegmatis, Fig. 8B) are distantly related to N. meningitidis (median ANI, <80%; Fig. 1).

Finally, we compared the number of inferred additional host species of PhiSpy predictions between vConTACT subclusters. Members of 1318_0 (including known prophages NgoΦ1 and NgoΦ2) have 3 to 5 additional host species, while members of other subclusters have 0 to 2 (Fig. S6, Table S2, tab D). Together, our findings suggest that diverse *Neisseria* species may be infected by the same phages.

10.1128/msystems.00083-22.6 FIG S6 Inferred additional host species of PhiSpy predictions belonging to each vConTACT subcluster. The distribution of the number of inferred additional host species is shown for dereplicated PhiSpy predictions that have a significantly higher CRISPR targeting density compared to the bacterial genome backbone. Interspecies CRISPR targeting data were used to infer additional host species for each phage. Data are grouped according to vConTACT subcluster. Download FIG S6, TIF file, 2.8 MB. Copyright © 2022 Orazi et al. 2022 Orazi et al. https://creativecommons.org/licenses/by/4.0/ This content is distributed under the terms of the Creative Commons Attribution 4.0 International license.

## DISCUSSION

In this study, we sought to broaden the diversity of phages known to infect *Neisseria* species. We used three different virus prediction tools to scan 248 genomes of commensal and pathogenic *Neisseria* species for prophages. Clustering approaches revealed that many of these predictions are dissimilar from known *Neisseria* MGEs (phages, plasmids, or the GGI) and phages described to infect other taxa. Therefore, we may have uncovered novel *Neisseria* phage diversity.

We also identified prophages in several commensal *Neisseria* species that are highly similar to the N. meningitidis prophages Pnm1 and -2 and MuMenB, as well as Mu-like phages that infect Haemophilus parasuis and Mannheimia haemolytica, two members of the *Gammaproteobacteria*. Although Pnm2 and MuMenB are defective, they may retain the ability to contribute genes to other coinfecting phages (59). In addition, we found several predicted *Neisseria* prophages that are highly similar to Mu-like phages that infect other *Betaproteobacteria*, Burkholderia cenocepacia and B. thailandensis.

Commensal *Neisseria* species frequently colonize the upper respiratory tracts of humans and animals. Thus, these species may encounter Haemophilus, *Mannheimia*, and *Burkholderia* species within these niches and could be exposed to the same or similar phages. Additionally, we found highly similar predicted prophages in different *Neisseria* species, including distantly related species.

CRISPR-Cas immune systems provide a historical record of encounters between microbes and MGEs. Previously, Zhang et al. identified CRISPR spacers that match the filamentous phage MDAΦ (44), and here, we found that 13 known *Neisseria* prophages are matched by spacers. To identify predictions that are more likely to be phages, we compared targeting of each predicted prophage to targeting of the backbone genome. We found that 20% of dereplicated predicted prophages (259/1,306) have a significantly higher targeting density than that of the backbone, and 74% of these (191/259) do not cluster with known *Neisseria* MGEs. Therefore, we believe that these 191 predictions warrant further study.

Moreover, we found evidence of widespread interspecies targeting of predicted prophages and backbone sequences by *Neisseria* spacers. We used those data to infer additional host *Neisseria* species of predictions that are significantly more highly targeted than the backbone. Building upon previous findings (36, 37), our results suggest that multiple known and predicted phages may be able to infect multiple species of *Neisseria*, including distantly related species.

In addition to defending against MGEs, CRISPR-Cas systems of N. meningitidis and other microbes may also play a role in restricting the exchange of chromosomal DNA between species (44, 45, 70, 71). Our observation that backbone sequences of various *Neisseria* species are targeted by N. meningitidis spacers at a low level is consistent with this model. Furthermore, multiple immune systems may work in concert to limit gene flow between *Neisseria* species (43, 72, 73).

This study has several important limitations. Commensal species are underrepresented among the available *Neisseria* genome assemblies and, thus, also in this study. This underrepresentation limits our ability to compare patterns of CRISPR targeting between species. Also, our inference of host species is limited by whether genomes encode CRISPR spacers (e.g., N. gonorrhoeae genomes do not encode CRISPR arrays).

We only used three virus prediction tools, among which only PhiSpy was specifically designed to predict prophages (i.e., integrated phages) (50). Our predictions are likely biased toward tailed phages; however, the diversity of *Neisseria* filamentous prophages has been explored recently (37).

Additionally, we do not know the true boundaries of the predicted prophages, whether they are intact or incomplete, and whether they are able to produce virions. Although only seven putative, active prophages were identified in the 51 genomes examined here, it is possible that additional prophages may be induced under certain conditions.

Prophages are known to influence the fitness and virulence of many bacterial species, including N. meningitidis (18–23). Furthermore, considerable evidence suggests that accessory genes are shared extensively between *Neisseria* species and that commensal species are a reservoir of antibiotic resistance and virulence genes (46, 74–78). Therefore, it is critical to understand the diversity and host range of phages, which have the potential to mobilize genes among *Neisseria* species and alter their evolutionary trajectories. Further research on phages is also crucial for developing phage therapy approaches (79).

By combining clustering and CRISPR targeting analyses, we have identified candidate, novel *Neisseria* phages and inferred that several may infect multiple species within this bacterial genus. We hope that our findings may inform future studies seeking to elucidate the impact of viruses on *Neisseria* biology. Finally, we believe that our work may have implications for understanding the interactions occurring among the diverse *Neisseria* species that colonize the oropharynx and the phages that infect them.

## MATERIALS AND METHODS

### Generation of bacterial genome data sets.

A set of high-quality bacterial genome assemblies was selected for prophage prediction. Specifically, *Neisseria* genome assemblies with *N*_50_ values of ≥250 kb and contigs of ≤10 were downloaded from GenBank (47, 48). This set of 248 assemblies is referred to as the “smaller *Neisseria* genome data set” (Table S1, tab A).

For the identification of CRISPR arrays, a second data set of bacterial genome assemblies was compiled as follows: *Neisseria* genome assemblies with an *N*_50_ value of ≥15 kb were downloaded from GenBank (47, 48) and limited to the species represented in the smaller genome data set. This second set of 2,619 assemblies is referred to as the “larger *Neisseria* genome data set” (Table S1, tab E).

All genome assemblies were downloaded on 30 March 2020. For each of the above-described data sets, the numbers of genomes corresponding to each *Neisseria* species are summarized in Table S1, tab F. These data sets include assemblies provided by the Wellcome Sanger Institute community resource project NCTC 3000 (https://www.sanger.ac.uk/resources/downloads/bacteria/nctc/) and the FDA-ARGOS genomic database resource (80).

### Construction of bacterial phylogenetic tree and heatmap of average nucleotide identity.

PubMLST (81) was used to concatenate sequences of the 53 genes encoding ribosomal proteins of each bacterial genome in the smaller *Neisseria* genome data set (82). Then, the concatenated protein sequences were used to create a maximum-likelihood ribosomal multilocus sequence typing (rMLST) tree using RAxML (83) (with the GTRCAT model and 100 bootstrap replicates), and it was visualized using iTOL v5 (84). The tree was rooted using midpoint rooting.

The pairwise average nucleotide identity (ANI) between bacterial genomes in the smaller genome data set was calculated using FastANI (85) and visualized as a heatmap using the R (86) package pheatmap (87).

### Prediction of prophages in *Neisseria* genomes.

To increase the likelihood of identifying novel *Neisseria* prophages, we selected three command-line tools that use different approaches to predict prophages in the smaller set of *Neisseria* genomes. PhiSpy (50) uses machine learning to search for characteristics that are unique to prophages (i.e., phages integrated in bacterial genomes), while VirSorter2 (51, 88) combines alignment and machine learning-based approaches to identify microbial viruses. PhiSpy and VirSorter2 both performed well when evaluated for their ability to predict prophages in bacterial genomes (56). We also selected Seeker (52), which uses deep learning to detect phages without relying on sequence features (e.g., genes or k-mers) to explore potential novel prophage diversity.

PhiSpy v4.2.19 was run in strict mode without HMM searches after training using a custom training set. To generate the custom set, we combined the PhiSpy default reference genomes with the N. gonorrhoeae genome FA 1090 (GenBank assembly version no. GCA_000006845.1) annotated with proteins from the dsDNA tailed phages (NgoΦ1-5) and filamentous phages (NgoΦ6-9) (29, 31). We did not add any N. meningitidis genomes to the training set because N. meningitidis MC58 (GCA_000008805.1) and N. meningitidis Z2491 (GCA_000009105.1) were already included in the reference data set.

VirSorter2 v2.1 was run using default settings, and Seeker was run using the prophage model (LSTM_type=“prophage”). The coordinates of each predicted prophage are provided in Table S2, tab A. The FASTA sequences of each predicted prophage are available at https://doi.org/10.6084/m9.figshare.19372802.

### Active prophage analysis.

The GenBank (47, 48) and SRA (48, 89) databases were searched for reads corresponding to each assembly in the smaller set of *Neisseria* genomes. Table S1, tab C contains a list of 51 assemblies that were found to have corresponding Illumina paired-end reads. Reads were retrieved from the SRA database using the prefetch and fasterq-dump tools from the SRA Toolkit (90). hafeZ v1.0.2 (55) was used to search for active prophages in each assembly in Table S1, tab C (using -T phrogs); results are available at https://doi.org/10.6084/m9.figshare.19372802. PropagAtE v1.1.0 (54) was used to estimate whether the 406 prophages predicted by PhiSpy, VirSorter2, and Seeker in the above-described assemblies are active or dormant (using -v to specify prophage coordinates).

### Dereplication of predicted prophages.

An all-by-all BLASTn (91) search was performed separately with prophages predicted by each tool. Predicted prophages were dereplicated at 95% length aligned using a custom script (blast_average_link_hier_clust_output_clusters.py; https://github.com/Alan-Collins/Neisseria-prophage-paper). Information about dereplicated predictions and the predictions used as their representatives is in Table S2, tab B. Known *Neisseria* plasmids were dereplicated using the same method.

### Hierarchical clustering of predicted prophages with *Neisseria* MGEs based on percent length aligned nucleotide sequence.

First, an all-by-all BLASTn (91) search was performed on dereplicated predicted prophages, dereplicated known *Neisseria* plasmids, and the gonococcal genetic island. Next, a distance matrix was created based on the percent length aligned (PLA) nucleotide sequence between pairs of MGEs (distance = 1 – PLA). The Python (92) package SciPy v1.6.1 (93) was then used to perform average-linkage clustering on the distance matrix. A custom script (identify_blast_clusters.py; https://github.com/Alan-Collins/Neisseria-prophage-paper) was used to extract cluster memberships and extract the tree in Newick format, which was visualized using iTOL v5 (84).

### vConTACT v.2.0 clustering of phages based on shared genes.

Prodigal v2.6.3 (94) was used to predict the protein-coding genes of known *Neisseria* phages and dereplicated predicted prophages (run in anonymous/metagenomic mode using -p meta). Afterward, Prodigal-generated protein sequences were clustered with reference viral genomes using vConTACT v2.0 (64, 66).

As reference viral sequences, we used viruses from the RefSeq database (48, 95) and 12,892 virus sequences provided by the Millard lab (96) (http://millardlab.org/bioinformatics/lab-scripts/supplementing-and-colouring-vcontact2-clusters/). Protein sequence files and mapping files generated on 30 May 2020 were downloaded on 20 May 2021. Clustering with reference sequences was performed separately on prophages predicted by each virus prediction tool. The following vConTACT settings were used: –rel-mode “Diamond”, –db “ProkaryoticViralRefSeq94-Merged” –pcs-mode MCL –vcs-mode ClusterONE. vConTACT networks were visualized in Cytoscape v3.8.2 (97) using the edge-weighted spring-embedded layout algorithm, which positions highly similar viruses close together. Duplicate edges were removed from the network, and reference viruses are only shown if they are connected by an edge to a known *Neisseria* prophage or predicted prophage.

### Analyses of vConTACT viral clusters.

We compared the similarity of predicted prophages to known viruses (i.e., known *Neisseria* phages and reference phages) between the three tools as follows. First, we examined each prediction’s connections to known viruses and identified the edge with the highest similarity score. Then, we compared the distributions of similarity scores between each tool using the Mann-Whitney U test implemented in the Python (92) package SciPy v1.7.3 (93).

To compare gene clusters between the members of each viral subcluster, Prokka v1.14.6 (98) was used to predict and annotate open reading frames (ORFs) of each phage (using the Pfam, TIGRFAM, and HAMAP databases) (99–101), and Clinker (102) was used to generate comparisons of annotated predicted proteins.

### Identification of CRISPR arrays in *Neisseria* genomes.

MinCED v0.4.2 (103) (using default settings) was used to identify CRISPR repeats in the smaller set of *Neisseria* genomes. For each species, the most common MinCED-identified repeat(s) was compared to the repeats included in the CRISPRCasDB website (104) (https://crisprcas.i2bc.paris-saclay.fr/MainDb/StrainList), and a consensus list of repeats was generated. The CRISPRCasTyper webserver (105) was used to predict the CRISPR subtype associated with each of the identified repeats.

Using the CRISPR repeats identified in high-quality genomes, we used a custom script (reps2spacers.py; https://github.com/Alan-Collins/Neisseria-prophage-paper) to run BLASTn (91) (using -task blastn-short) and process results to identify spacers in the larger set of *Neisseria* genomes. To investigate sharing of identical spacers between genomes of different species, an all-by-all BLASTn (91) search (using -task blastn-short) was performed on all spacers that were identified in the larger *Neisseria* genome data set.

### Prediction of CRISPR targeting of prophages and bacterial genomes.

BLASTn (91) (using -task blastn-short) was used to identify matches between *Neisseria* CRISPR spacers (identified in the larger *Neisseria* genome data set) and either prophages or high-quality bacterial genomes. Matches were filtered as follows: the spacer had to match the target with 100% identity over the entire length of the spacer (i.e., 0 mismatches). Additionally, matches between spacers and CRISPR arrays found in bacterial genomes or prophages were removed. CRISPRopenDB (69) was used to predict CRISPR targeting of predicted prophages by other bacterial taxa (using the default setting of 2 mismatches).

### Comparison of CRISPR targeting of prophages versus bacterial genome backbones and statistical testing.

To compare the CRISPR targeting of predicted prophages and the genomes in which they were found (i.e., the backbone), we used the following method. As described above, matches between CRISPR spacers and targets in prophages or bacterial genomes were identified using BLASTn (91) (using -task blastn-short), and only hits with 100% identity over the full length of the spacer were kept. CRISPR spacers were excluded if they matched a CRISPR array found in either a predicted prophage or a bacterial genome.

Next, we quantified CRISPR targeting per kb; importantly, this was done differently for prophages and backbones as follows. For prophages, the targeting density is the number of CRISPR matches divided by the prophage length in kb. For backbones, targeting is the number of matches in the entire bacterial genome minus any matches in locations that are known/predicted to be part of a prophage and locations that are part of CRISPR arrays; the length is calculated by subtracting the length of all prophages identified in the genome from the length of the entire bacterial genome. Then, the backbone targeting density was calculated as the number of CRISPR targets (not in a known/predicted prophage or CRISPR array) divided by the length of the genome minus the lengths of all known/predicted prophages.

Afterward, we performed statistical testing to test whether there is a difference between the targeting density of the prophage and the backbone for each prophage; to do this, each kb of prophage or bacterial genome was treated as a separate datapoint. Specifically, we performed a Mann-Whitney U test to compare each of the CRISPR targeting counts for the separate kb bins between each prophage and the backbone using the Python (92) package SciPy v1.7.3 (93). The *P* values for all phages tested were adjusted with the Holm-Sidak correction using the Python (92) package Statsmodels v0.13.0 (106).

### Inferring host bacterial species of known and predicted prophages.

Results from the interspecies CRISPR targeting analysis described above were used to infer the additional host species of known *Neisseria* phages and dereplicated predictions made by PhiSpy, VirSorter2, and Seeker. Only dereplicated predicted prophages that were found to have a significantly higher targeting density compared to the rest of the genome in which they were identified (as described above) were included in this analysis. Any species that was found to target a prophage with ≥1 spacer was inferred to be a host of that prophage (in addition to the species in which the prophage was identified).

### Data availability.

All of the genome sequences used in this study were downloaded from GenBank. The accession numbers of the 248 bacterial genome assemblies in the high-quality, smaller set of genomes are provided in Table S1, tab A. The accession numbers of all 2,619 bacterial genome assemblies used in this study are included in Table S1, tab E. The accession numbers of phage and plasmid genomes are listed in Table S1, tabs B and D, respectively.

The custom scripts created for analysis of data in this study are available at https://github.com/Alan-Collins/Neisseria-prophage-paper. The data sets generated in this study are provided in the supplemental material and are also available at https://doi.org/10.6084/m9.figshare.19372802.
