# Supplementary figures and images for: Prediction of Prophages and Their Host Ranges in Pathogenic and Commensal Neisseria Species
Source: mSystems. 2022 Apr 14;7(3):e00083-22. doi: 10.1128/msystems.00083-22 (PMC9238386; doi:10.1128/msystems.00083-22)

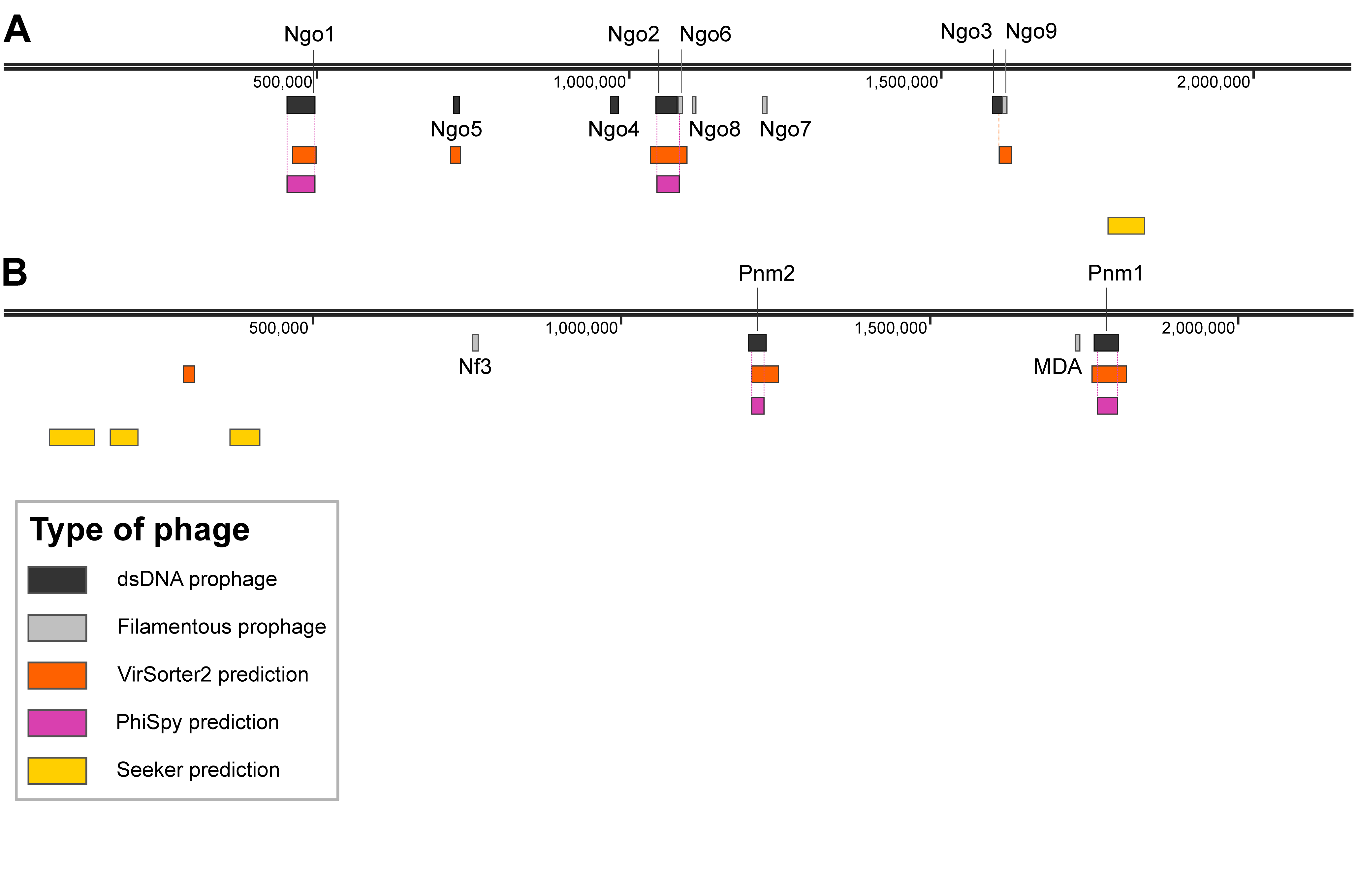

Supplement: FIG S1 [file msystems.00083-22-sf001.tif]

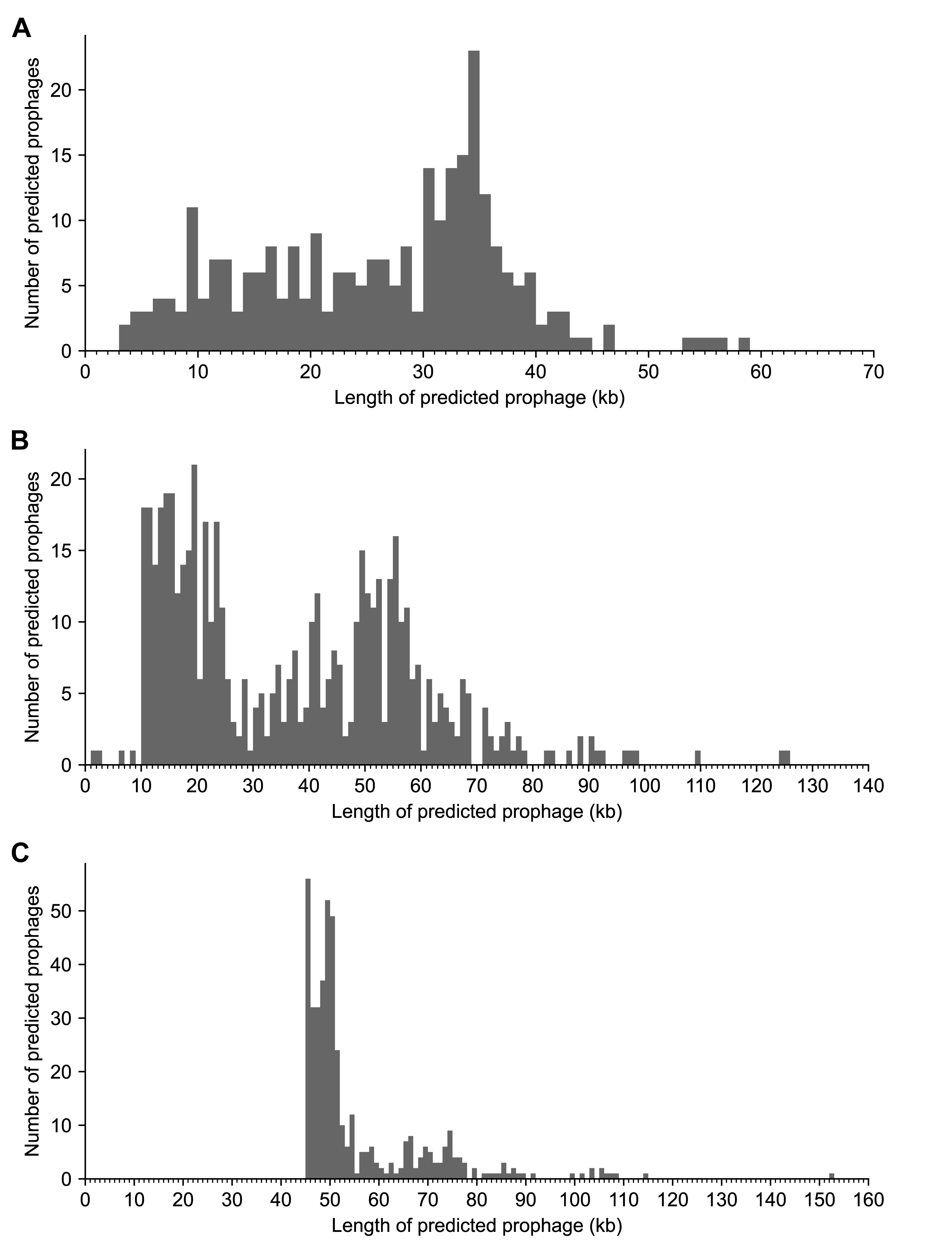

Supplement: FIG S2 [file msystems.00083-22-sf002.tif]

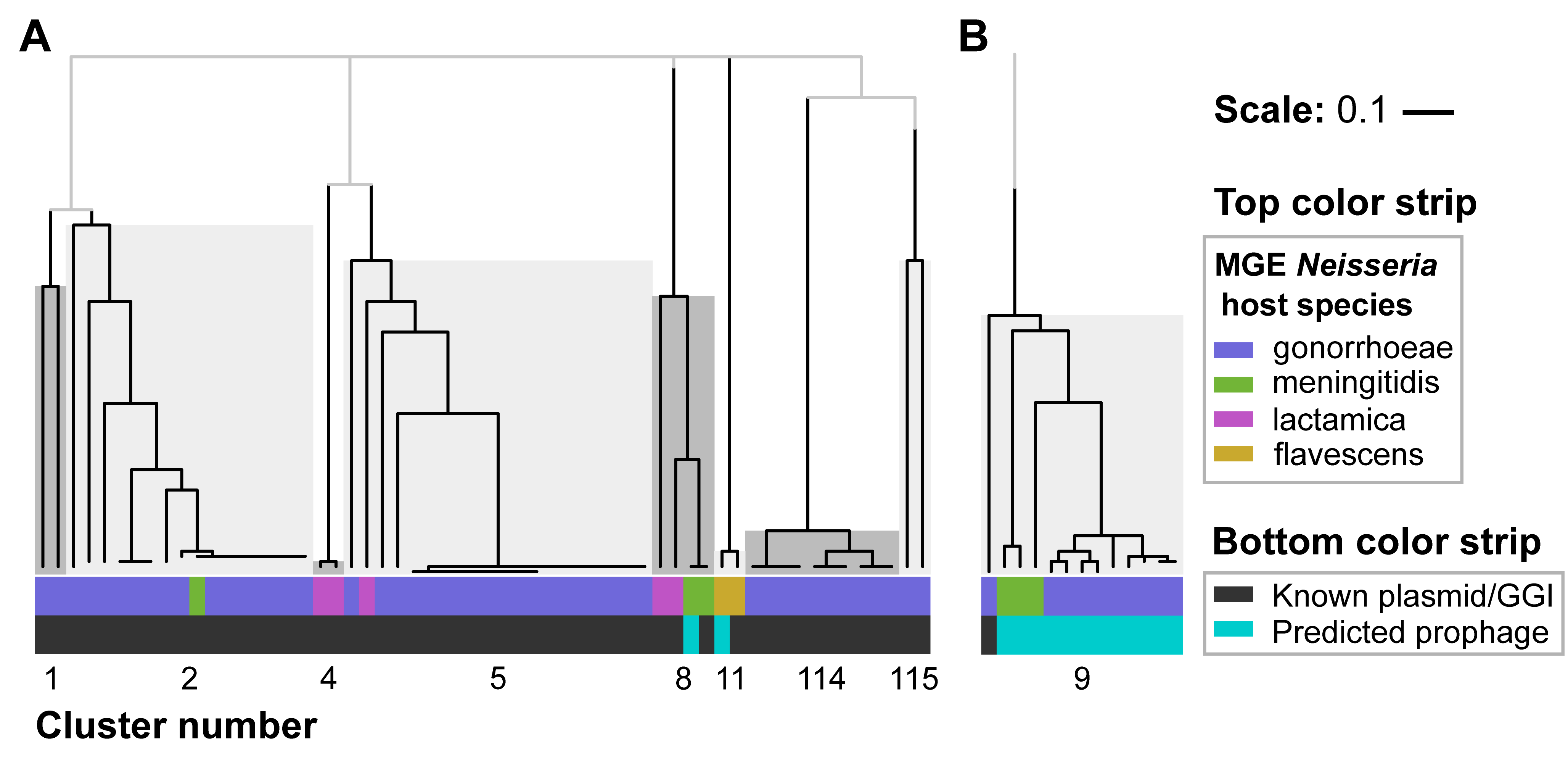

Supplement: FIG S3 [file msystems.00083-22-sf003.tif]

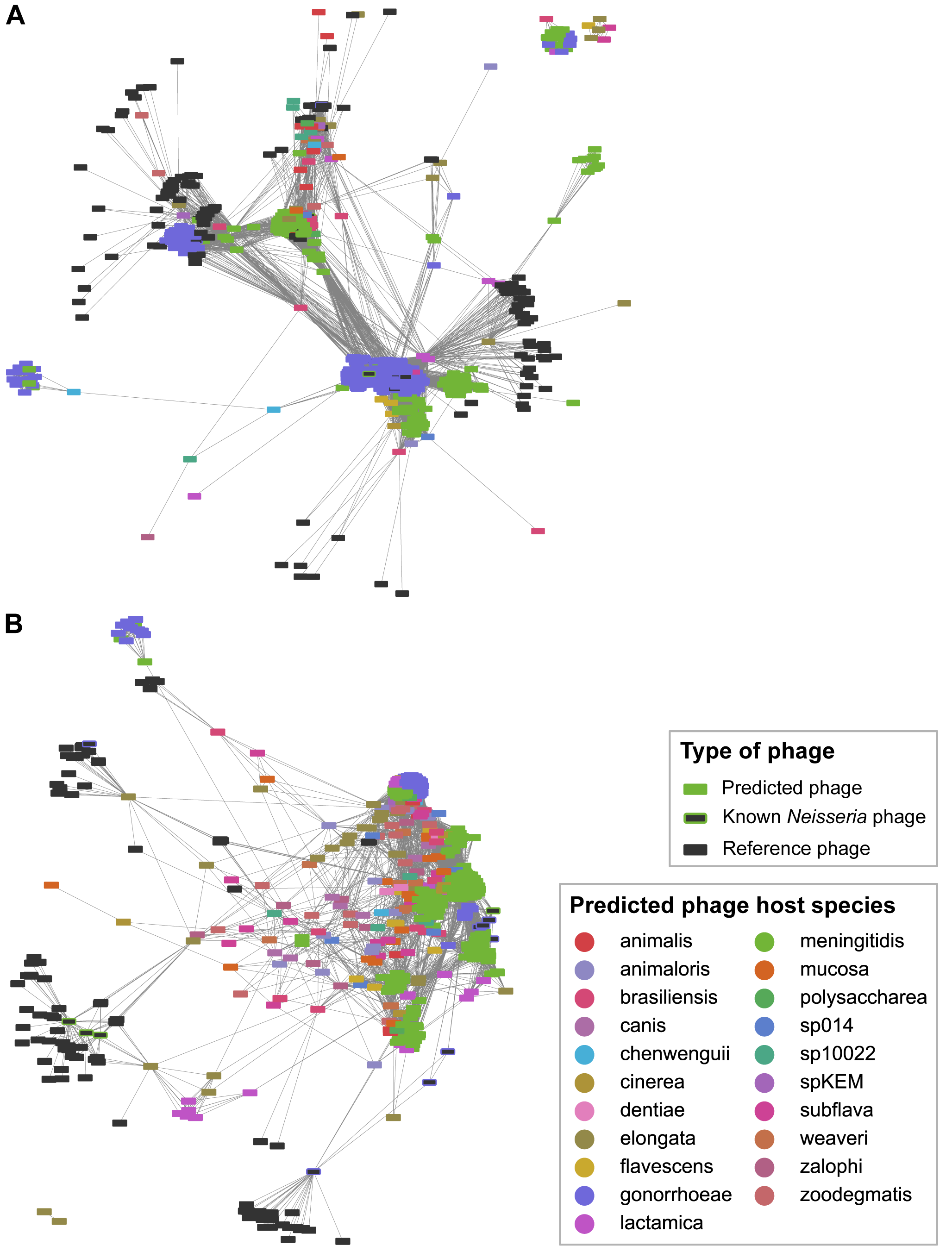

Supplement: FIG S4 [file msystems.00083-22-sf004.tif]

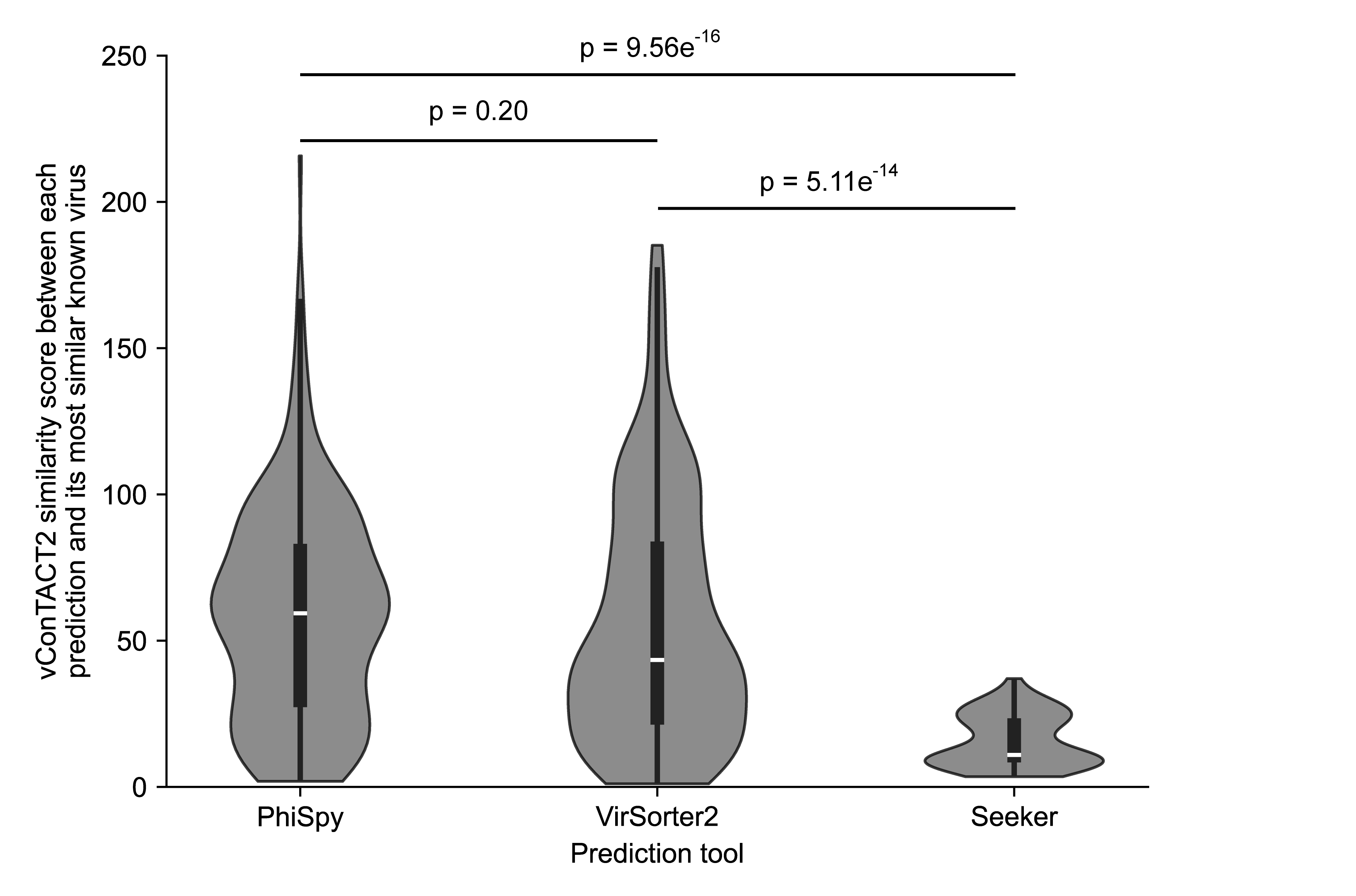

Supplement: FIG S5 [file msystems.00083-22-sf005.tif]

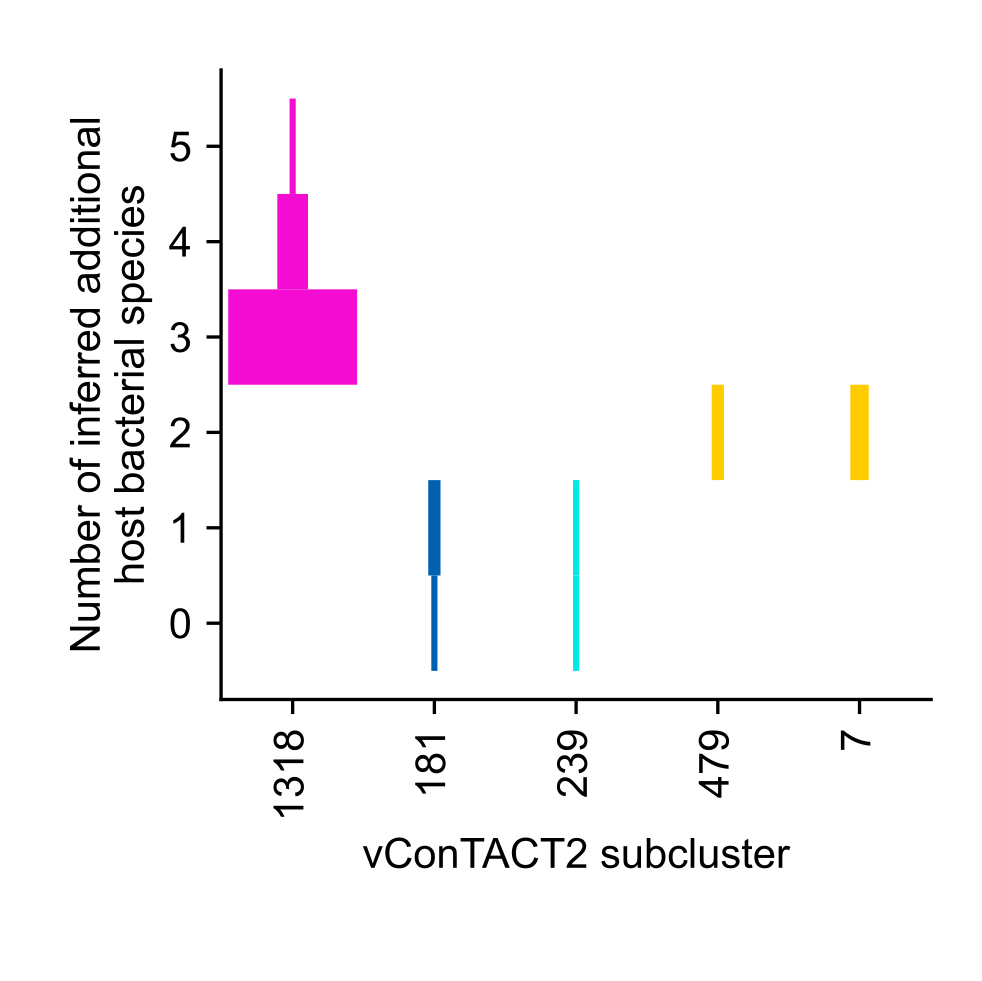

Supplement: FIG S6 [file msystems.00083-22-sf006.tif]
